# Xenografted human iPSC-derived neurons with the familial Alzheimer’s disease *APP*^*V717I*^ mutation reveal dysregulated transcriptome signatures linked to synaptic function and implicate LINGO2 as a disease signaling mediator

**DOI:** 10.1007/s00401-024-02755-5

**Published:** 2024-06-25

**Authors:** Wenhui Qu, Matti Lam, Julie J. McInvale, Jason A. Mares, Sam Kwon, Nelson Humala, Aayushi Mahajan, Trang Nguyen, Kelly A. Jakubiak, Jeong-Yeon Mun, Thomas G. Tedesco, Osama Al-Dalahmah, Syed A. Hussaini, Andrew A. Sproul, Markus D. Siegelin, Philip L. De Jager, Peter Canoll, Vilas Menon, Gunnar Hargus

**Affiliations:** 1 Department of Pathology and Cell Biology, Presbyterian Hospital, Columbia University, 650W 168th Street, New York, NY USA; 2 Department of Neurology, Center for Translational and Computational Neuroimmunology, Neurological Institute, Columbia University, 710 West 168th Street, New York, NY USA; 3 Department of Neurosurgery, Columbia University, New York, NY USA; 4 Taub Institute for Research on Alzheimer’s Disease and the Aging Brain, Columbia University, New York, NY USA

**Keywords:** Alzheimer’s disease, Induced pluripotent stem cells, iPSCs, Transplantation, Disease modeling, Grafts, Single-nucleus RNA sequencing, snRNA-seq

## Abstract

Alzheimer’s disease (AD) is the most common cause of dementia, and disease mechanisms are still not fully understood. Here, we explored pathological changes in human induced pluripotent stem cell (iPSC)-derived neurons carrying the familial AD *APP*^*V717I*^ mutation after cell injection into the mouse forebrain. *APP*^*V717I*^ mutant iPSCs and isogenic controls were differentiated into neurons revealing enhanced Aβ_42_ production, elevated phospho-tau, and impaired neurite outgrowth in APP^V717I^ neurons. Two months after transplantation, APP^V717I^ and control neural cells showed robust engraftment but at 12 months post-injection, APP^V717I^ grafts were smaller and demonstrated impaired neurite outgrowth compared to controls, while plaque and tangle pathology were not seen. Single-nucleus RNA-sequencing of micro-dissected grafts, performed 2 months after cell injection, identified significantly altered transcriptome signatures in APP^V717I^ iPSC-derived neurons pointing towards dysregulated synaptic function and axon guidance. Interestingly, APP^V717I^ neurons showed an increased expression of genes, many of which are also upregulated in postmortem neurons of AD patients including the transmembrane protein LINGO2. Downregulation of LINGO2 in cultured APP^V717I^ neurons rescued neurite outgrowth deficits and reversed key AD-associated transcriptional changes related but not limited to synaptic function, apoptosis and cellular senescence. These results provide important insights into transcriptional dysregulation in xenografted APP^V717I^ neurons linked to synaptic function, and they indicate that LINGO2 may represent a potential therapeutic target in AD.

**Supplementary Information:**

The online version contains supplementary material available at 10.1007/s00401-024-02755-5.

## Introduction

Alzheimer’s disease (AD) is the most frequent form of dementia affecting millions of people without a cure. AD is characterized by deposition of β-amyloid plaques and phosphorylated tau (p-tau)-positive neurofibrillary tangles in patient brains with neuronal loss, synaptic dysfunction and gliosis [56, 57]. Mutations in genes involved in Aβ production, including *APP*, *PSEN1*, and *PSEN2*, cause early-onset familial AD (fAD) [12, 25], and genome-wide association studies (GWAS) have identified gene variants, such as *APOE*, that are associated with increased risk of developing sporadic late-onset AD [75].

Given the limited treatment options for AD patients and the growing demand for disease modeling platforms using human cells, induced pluripotent stem cells (iPSCs) have emerged as a powerful tool to examine AD phenotypes. Differentiated fAD neurons show AD pathological features in vitro including increased Aβ_42_ production and elevated levels of p-tau [29, 35, 41, 45, 50, 64, 77]. However, adequate cell–cell interactions and the systematic complexity of the brain microenvironment are difficult to model in vitro. The injection of diseased or healthy iPSC-derived neural cells into the mouse brain could overcome this limitation, since previous reports have demonstrated successful survival of human iPSC-derived neural cells after intracranial injection into rodent brains [2, 15, 16, 21, 22, 28, 42, 61]. These models provide unique opportunities to characterize neuronal maturation, cell type-specific vulnerability or dysregulation, and graft–host interactions in a more physiological environmental context in vivo, as compared to pure in vitro studies.

Here, we injected iPSC-derived neural precursor cells (NPCs) carrying the fAD-associated *APP*^*V717I*^ mutation (*APP*^*Lon*^) and their isogenic controls (Ctrls) into the striatum and cortex of 2-month-old, immunocompromised mice, followed by histological and transcriptional analyses on grafted cells. This approach generated neuronal grafts with significantly altered transcriptome profiles in *APP*^*V717I*^ neurons that recapitulate synaptic function-related transcriptomic dysregulation of AD with identification of potential therapeutic targets, including the transmembrane protein LINGO2, as demonstrated by comparative single cell analysis using single nucleus RNA-sequencing (snRNA-seq).

## Materials and methods

### iPSC generation and differentiation

CRISPR/Cas9 genome editing was used to generate independent *APP*^*V717I*^ heterozygous knock-in clones in the control iPSC line IMR90 [clone 4, WiCell; 26, 78, 79] as described previously for clone 88 [68]. Clone C20 (red dots) and clone C30 (blue dots) were utilized in this study. These clones also harbor a tet-on NGN2 construct (with rtTA), but doxycycline was not added to differentiation steps. NPCs were generated from iPSC lines as previously described [14, 20, 22, 52, 53]. Briefly, ReLeSR (STEMCELL 05872) dissociated iPSC colonies were resuspended in iPSC media containing 10 µM SB-431542 (Ascent Scientific), 1 µM Dorsomorphin, 3 µM CHIR 99021 (Axon Medchem), and 0.5 µM purmorphamine (PMA; Alexis) and cultured as embryoid bodies (EBs). Medium was changed on day 2 to NPC priming medium containing the same supplements in the base of equal mix of DMEM-F12 (Gibco) and Neurobasal A (Gibco) supplemented with 0.5X B27 without vitamin A (Gibco; 12587010), 0.5X N2 (Gibco, 17-502-048), and 1% penicillin/streptomycin/glutamine (N2/B27 medium). On day 4, SB-431542 and Dorsomorphin were replaced with 200 µM ascorbic acid as the NPC maintenance media. On day 6, EBs were mechanically dissociated into smaller pieces and plated onto Matrigel-coated plates (Corning Matrigel membrane matrix HC 08-774-392) in NPC maintenance media. On day 12, cells were dissociated with Accutase (Millipore Sigma) into single cells and replated onto new Matrigel-coated plates. NPC lines were expanded and banked.

All experiments in this study used NPCs passages above 12. NPCs were differentiated into neurons as previously described [14, 22, 53]. Briefly, NPCs were dissociated with Accutase into 50,000 cells/mL and plated onto new Matrigel-coated plates with NPC maintenance media, which is changed to neuronal induction N2/B27 medium that contains 10 ng/mL FGF8, 1 µM PMA, and 200 µM ascorbic acid 2 days later. On day 6 of neuronal induction, media was replaced by neuronal maturation media containing 10 ng/mL GDNF, 10 ng/mL TGF-β3, 10 ng/mL BDNF (Peprotech), 200 µM ascorbic acid and 500 µM dbcAMP (Sigma Aldrich, D0260). Media was changed every other day and cells were harvested after 3 weeks in neuronal maturation media.

### Transplantation

All animal experiments were adhered to protocols approved by the Animal Care and Use Committee at Columbia University (AC-AABM1557). Transplantation experiments were conducted as previously described [22]. Briefly, cultured NPCs were lifted using Accutase (Millipore Sigma) and washed twice with PBS then resuspended in PBS at a density of 50,000 cells/µL. Three microliters of cell suspension were injected into the right hemisphere (position from bregma: + 0.5 mm anterior, + 2.0 mm lateral, − 3.0 mm ventral) of 8-week-old NSG mice (NOD.Cg-Prkdc^scid^ Il2rg^tm1Wjl^/SzJ mice, Strain #: 005557, The Jackson Laboratory).

### Microdissection of grafts

Mice were anesthetized with ketamine/xylazine cocktail (ketamine concentration: 0.08 mg/g of mouse; xylazine concentration: 0.012 mg/g of mouse) followed by decapitation. Brains were dissected out and sectioned into 400 μm brain slices in ice-cold HBSS using a tissue chopper (McIlwain). Brain slices were then transferred into HBSS-filled petri dishes and human grafts were micro-dissected under a dissection microscope (Morrell) using 21-gauge needles (BD™). Dissected grafts were snap-frozen and stored at − 80 °C for snRNA-seq or protein isolation.

### Immunohistochemistry of mouse brain tissue with grafts

Mice were anesthetized with ketamine/xylazine cocktail and perfused with PBS followed by 4% PFA 2 months after transplantation. Brains were harvested and fixed in 4% PFA overnight followed by storage in 15% sucrose in PBS for at least 24 h till sectioning. Brains were snap-frozen in cold 2-methyl-butane (MilliporeSigma) on dry ice and sectioned using a cryostat (Leica) at 10 µm thickness. Sections were mounted onto glass slides for hematoxylin and eosin (H&E) staining or immunofluorescent staining as previously described with a few modifications [51]. Briefly, frozen slides were warmed to room temperature then washed in PBS for three times followed by 1 h blocking in 5% normal goat serum (NGS) or normal donkey serum (NDS) in 0.2% PBST (Triton X100). Primary antibodies were diluted in blocking buffer according to manufacturer’s suggested dilutions and were put onto slides in a humidified staining tray (Research Products International Corp; 50-998-211) overnight. On the following day, slides were washed in PBS three times and incubated in secondary antibodies conjugated with AlexaFluor (Invitrogen) for 1 h at room temperature, followed by three times of washes with PBS and mounting in antifade mounting medium with DAPI (Vector Laboratories; NC9029229). Images of grafts were taken using the Leica Thunder microscope. Primary antibodies used in this study include: HNA (human nuclear antigen; MilliporeSigma; MAB1281; 1:200), NeuN (Abcam; ab104225; 1:500); LINGO2 (Thermo Fisher; PA5-99869; 1:200); hNCAM (human-specific NCAM; ERIC1; Santa Cruz; sc-106; 1:200); hGFAP (human-specific GFAP; Takara Bio; STEM123; 1:500), GFAP (MilliporeSigma; MAB360; 1:500), IBA1 (Wako; 1919741; 1:500), and SPP1 (R&D; AF808SP; 1:50).

### Quantification of graft size

H&E-stained brain slides were scanned using the Leica Thunder microscope. Unbiased estimates of the volume of transplants were calculated using the Cavalieri estimator probe on H&E-stained serial 10 μm-thick brain sections. Cell counts were obtained applying the fractionator probe. Every tenth 10-μm-thick section of the graft was analyzed for the quantification. For immunofluorescent stained sections, grafts were located based on HNA^+^ area or cellular density of DAPI. HNA^+^, DAPI^+^, NeuN^+^, Iba1^+^, and SPP1^+^ cells were manually counted. GFAP immunointensity within the graft and within 50 μm diameter surrounding the graft was measured using ImageJ software. QuPath software was used for the quantification of hNCAM^+^ fibers in indicated brain areas.

### Single nuclei isolation and sequencing

Nuclei from micro-dissected grafts were isolated as previously described [3]. Briefly, frozen grafts were homogenized using a Dounce homogenizer in a tissue homogenizing buffer that contains 30% sucrose and 0.1% Triton-X 100. 15 strokes with the loss pestle followed by 15 strokes with the tight pestle were applied to each graft. The brain lysate was then mixed well using P1000 pipette followed by filtration through 35 μm nylon mesh (Corning; 352235), followed by 10 min centrifugation of 1000 g at 4 °C. The pellet was resuspended in homogenizing buffer and filtered through 35 μm nylon mesh again followed by 10 min centrifugation of 1000*g* at 4 °C. The pellet was resuspended in PBS supplemented with 1% BSA and a RNAase inhibitor (Invitrogen; SUPERaseIN; AM2696). Samples were then submitted to the Columbia Genome Center for further processing and sequencing. Chromium Controller (10× Genomics) was used and single Cell 3′ Reagent Kit v2 or v3 (Chromium Single Cell 3’ Library & Gel Bead Kit v2, catalog number: 120237; Chromium Single Cell A Chip Kit, 48 runs, catalog number: 120236; 10× Genomics) was used. Illumina NOVAseq 6000 platformV4 with 150 bp paired end reads was applied for the sequencing step.

### Statistics

Statistical comparisons between groups were performed using GraphPad Prism. Unpaired two-tailed t-test was used for comparing two groups as indicated in the figure legends. One-way ANOVA followed by Tukey’s multiple comparison tests was used to compare more than two groups.

### Data availability

The raw fastq data of bulk and single nucleus RNA-seq data are available through GEO under the SuperSeries record of GSE231640 or individually through GEO accession numbers GSE231638 and GSE231639, respectively.

Additional information about materials and methods used in this study is provided as supplemental material.

## Results

### APP^V717I^ iPSC neurons show amyloid and tau pathology, impaired neurite outgrowth, and dysregulated transcriptional and metabolic signatures

**Fig. 1:**
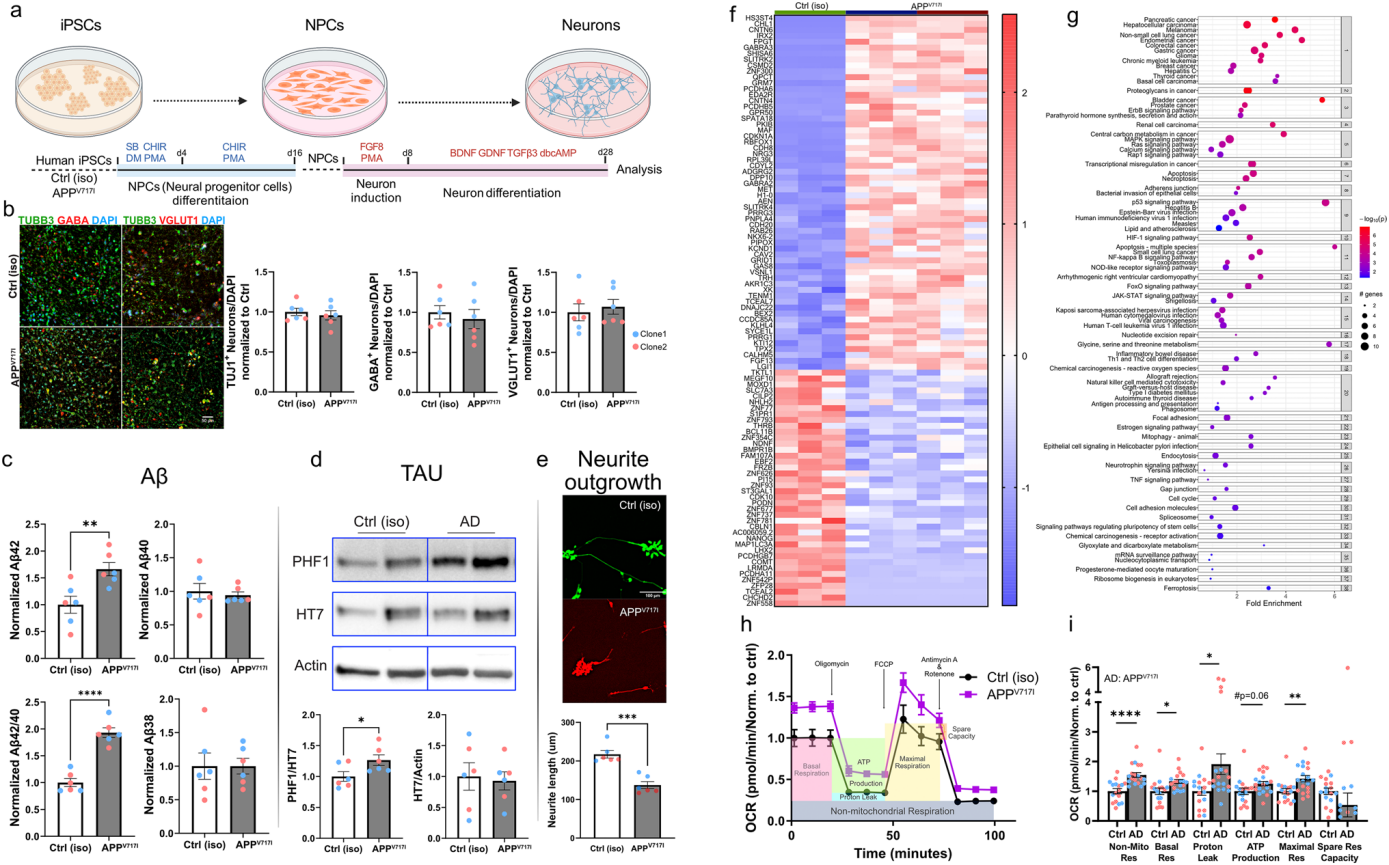
APP^V717I^ iPSC neurons show amyloid and tau pathology, impaired neurite outgrowth, and dysregulated transcriptional and metabolic signatures. **a** Schematic illustration of neuronal differentiation in vitro. **b** Immunostainings and quantification of βIII-tubulin^+^ (TUBB3^+^, TUJ1^+^), GABA^+^ and vGLUT1+ neurons. Nuclei are counterstained with DAPI. APP^V717I^ and Ctrl neurons show equivalent numbers of neurons as well as glutamatergic and GABAergic neuronal subtypes. Blue and red dots represent the two different cell clones, and each dot represents an independent differentiation. **c** Amyloid β ELISA for Aβ_42_, Aβ_40_ and Aβ_38_ on conditioned media of APP^V717I^ and Ctrl neurons showing increased production of Aβ42 compared with Ctrls, driving the increase of Aβ42/Aβ40 ratio. No differences in Aβ40 and Aβ38 levels were observed between APP^V717I^ and Ctrl. **d** Western blot images and quantification of the expression of PHF1 (p-tau), HT7 (total tau) and ACTIN in APP^V717I^ and Ctrl neurons showing increased p-tau but no changes in total tau expression in APP^V717I^ neurons. **e** Representative images and quantifications of neurite outgrowth in APP^V717I^ and Ctrl neurons labeled with mCherry and GFP, respectively. APP^V717I^ neurons show an impairment of neurite outgrowth 5 days of post-differentiation. **f** Heatmap of the top 100 most variable DEGs in neurons from bulk RNA-seq that are similar between the two APP^V717I^ clones but are significantly different from Ctrls. **g** Active network pathway enrichment analysis of DEGs in APP^V717I^ neurons and Ctrl neurons show changes of many cellular pathway in APP^V717I^ neurons. The x-axis shows the normalized enrichment values. The adjusted *p* value and number of genes are also denoted by color and by size, respectively. **h** Seahorse assays of APP^V717I^ and Ctrl neurons showing that APP^V717I^ neurons have an elevated oxygen consumption rate compared to Ctrl neurons. **i** Quantification of seahorse assays. Student *t*-test was used in **b**, **c** and **i**. *p < 0.05, **p < 0.01, ***p < 0.001, ****p < 0.0001

Two independent iPSC clones carrying the *APP*^*V717I*^ mutation and their isogenic Ctrls were programmed into stable neural progenitor cell (NPC) lines, followed by further differentiation into neurons for 4 weeks (Fig. 1a). Immunofluorescent staining showed successful generation of neurons expressing neuron-specific class III beta-tubulin (TUBB3^+^; TUJ1^+^). *APP*^*V717I*^- and Ctrl-derived NPCs demonstrated equivalent differentiation into glutamatergic and GABAergic neurons (Fig. 1b). Aβ ELISA of the media supernatant revealed elevated Aβ_42_ secretion in *APP*^*V717I*^ neurons, driving the increase of Aβ_42_/Aβ_40_ ratio without changes in the Aβ_40_ and Aβ_38_ levels (Fig. 1c). An analysis of the overall expression of the APP protein did not show a significant difference between the two groups, implying that the elevated production of Aβ_42_ may be attributed to APP cleavage rather than an increase in APP expression (Figure S1a, b). This observation aligns more closely with the physiological characteristics observed in human AD cases [44]. APP^V717I^ neurons expressed increased levels of p-tau assessed by elevated PHF1 (Ser396/Ser404) antibody reactivity (Fig. 1d), while other changes in p-tau expression were not observed, including Ser202/Ser205 (AT8), S356 (p-Tau 356), T217 (p-Tau 217), and T231 (AT180) (Figure S1a, b). To visualize neurites, Ctrl or APP^V717I^ NPCs were labeled with GFP or mCherry, respectively, and neurite length was evaluated in neurons 5 days of post-differentiation. An assessment of neurite lengths in APP^V717I^ and Ctrl cultures revealed an impairment of neurite outgrowth in the APP^V717I^ group (Fig. 1e).

Next, we performed bulk RNA-seq on differentiated APP^V717I^ and Ctrl neurons (Fig. 1f and g). We identified the top 100 most variable genes conserved between APP^V717I^ neurons derived from two separate clones, and significantly different from their isogenic Ctrls (Fig. 1f; Table S1). Several top differential genes are involved in synaptic regulation including *GABRA3*, *GRM7*, *RBFOX1*, and *DPP10* [30, 33, 62, 69, 73]. Active sub-pathway enrichment analysis revealed enrichment of several AD-related pathways including the MAPK signaling pathway, calcium signaling pathway, apoptosis, and the p53 signaling pathway (Fig. 1g; Table S1). Interestingly, several cancer-related pathways were also significantly dysregulated in APP^V717I^ cells. We also compared our in vitro bulk-seq DEGs with published in vitro sequencing data on human *PSEN* mutation carrier-derived iPSC neurons [11]. While we did not identify transcriptome profiles of dedifferentiation [11], a feature also seen in directly transdifferentiated AD-induced neurons (AD iNs) [37], we observed dysregulation of some cell-cycle reentering makers in our APP^V717I^ neurons including *CDKN1A*, *TP53*, *CDK1*, *HES1* and REST-repressed (*GAD1*) gene. Pathway analysis of overlapping up- and downregulated genes in PSEN and APP^V717I^ neurons showed some common significantly dysregulated pathways including cancer-related pathways and cellular senescence (Figure S1c, d), suggesting convergent dysregulated pathways affected by familial *PSEN* and *APP* mutations.

Next, we wondered if APP^V717I^ neurons show mitochondrial phenotypes since mitochondrial dysfunction and abnormal bioenergetic profiles have been implicated in the pathogenesis of AD, including elevated oxidative stress [40]. Our bulk expression data identified dysregulation of metabolic and mitochondrial function-related pathways in APP^V717I^ neurons, including central carbon metabolism, amino acid metabolism and calcium signaling (Fig. 1g). We evaluated mitochondrial function in APP^V717I^ neurons. Seahorse assays revealed an increased oxidative consumption rate in APP^V717I^ neurons with elevated basal respiration, proton leak, maximal respiration, non-mitochondrial respiration, and a trend towards increased ATP production (*p* = 0.06) (Fig. 1h and i). We then performed polar metabolite profiling of metabolites involved in the glycolysis, TCA cycle and the metabolism of fatty acids, amino acids, and nucleotides in our APP^V717I^ neurons combined with quantitative pathway enrichment analysis of dysregulated metabolites. These analyses revealed alterations in several key metabolic pathways in APP^V717I^ neurons, including glycolysis, aspartate metabolism, urea cycle and purine and pyrimidine metabolism, which have been implicated in AD (Figure S1e-f; Table S1) [47]. These data indicate altered bioenergetic profiles in APP^V717I^ neurons compared to their isogenic Ctrls.

### APP^V717I^ and Ctrl iPSC-derived neural grafts survive in the adult mouse brain and are composed predominantly of neurons

**Fig. 2:**
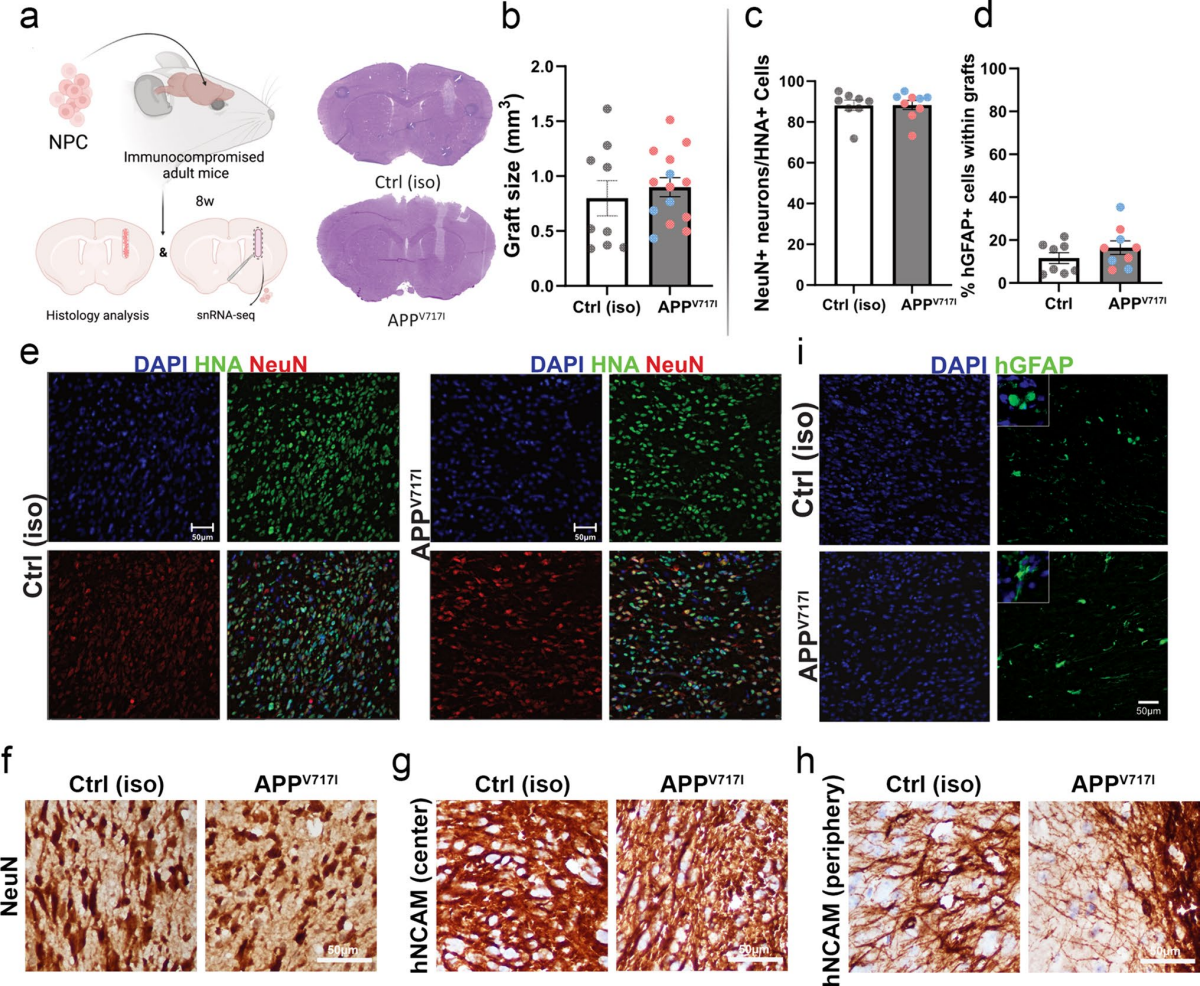
Histology of APP^V717I^ and Ctrl grafts 2 months after cell injection into the cortex and striatum of adult mice. **a** Schematic illustration of NPC transplantation into the brain of an immunocompromised adult mouse. Post-transplantation analysis, including histology and snRNA-seq, was conducted after 8 weeks of grafting. **b** H&E staining and volume quantification of mouse brain slices with human grafts. Human cells appear lighter compared to mouse cells. Graft sizes were comparable between APP^V717I^ and Ctrl. **c** and **d** Quantification of neurons (**c**) and astrocytes (**d**) in APP^V717I^ and Ctrl grafts. Around 90% human cells differentiated into neurons and 10% of cells differentiated into astrocytes in both groups. **e** Immunostainings of Ctrl and APP^V717I^ grafts for neurons using antibodies for NeuN and human nuclear antigen (HNA) with DAPI nuclear staining. **f** DAB stainings for NeuN highlighting neurons within Ctrl and APP^V717I^ grafts. **g** and **h** DAB stainings for the neural cell adhesion molecule NCAM using a human-specific antibody (hNCAM) highlighting neurons in the center (**g**) and periphery (**h**) of Ctrl and APP^V717I^ grafts. **i** Immunostainings of Ctrl and APP^V717I^ grafts for astrocytes using an antibody for human glial fibrillary acidic protein (hGFAP) with DAPI nuclear staining.

To study the pathology of APP^V717I^ neurons in a three-dimensional, physiological environment, we next transplanted APP^V717I^ and Ctrl iPSC-derived NPCs into the cortex and striatum of 2-month-old immunodeficient NOD.Cg-*Prkdc*^*scid*^* Il2rg*^*tm1Wjl*^/SzJ mice (Fig. 2a). Eight weeks after injection, brains were examined histologically, revealing viable neuronal grafts in all animals without tissue overgrowth and with similar graft sizes in the APP^V717I^ and Ctrl group (Fig. 2b). Quantification of immunostainings for NeuN, human-specific GFAP and human nuclear antigen (HNA) demonstrated that about 90% of the injected human cells had differentiated into NeuN^+^/HNA^+^ neurons and that about 10% had differentiated into hGFAP^+^ astrocytes, with similar percentages in both groups (Fig. 2c–i). APP^V717I^ and Ctrl neurons also expressed the neural cell adhesion molecule NCAM using a human-specific antibody (hNCAM; Fig. 2g and h). These findings indicated successful survival and maturation of neural cells in vivo (Fig. 2b). No Aβ^+^ amyloid plaques or p-tau^+^ neurofibrillary tangles were observed at this point of analysis. We also examined the host microenvironment within and adjacent to the APP^V717I^ and Ctrl neuronal grafts and quantified the abundance of GFAP^+^ reactive astrocytes and of Iba1^+^ and SPP1^+^ microglia in the graft core and at the graft–host interface. Although we did not observe significant differences in microglial activation and astrocyte reactivity, a trend towards increased GFAP reactivity within AD grafts (*p* = 0.06) was present in these areas at this time point of grafting (Figure S2a, b).

### Single-nucleus RNA-seq reveals increased vulnerability and synaptic dysfunction in transplanted APP^V717I^ neurons

**Fig. 3:**
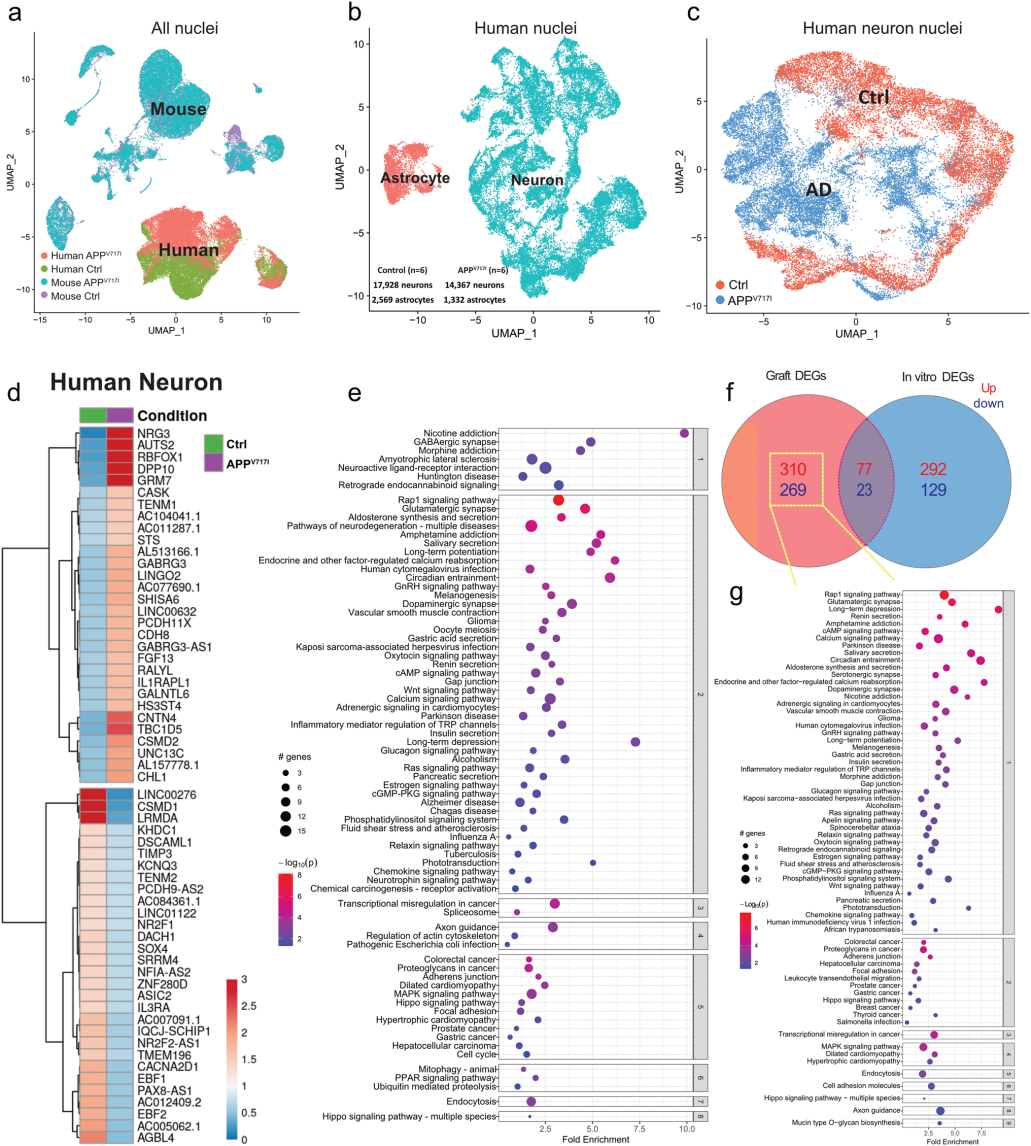
Single-nucleus RNA-sequencing of APP^V717I^ and Ctrl grafts 2 months post-engraftment. **a**–**c** UAMPs showing separations of nuclear profiles comparing human versus mouse (**a**), human neurons versus human astrocytes (**b**), as well as human APP^V717I^ neurons versus human Ctrl neurons (**c**). **d** Heatmap showing the top 30 most variable up- and downregulated DEGs in grafted APP^V717I^ versus Ctrl neurons. **e** Pathway enrichment analysis of DEGs in grafted APP^V717I^ versus Ctrl neurons. The *x*-axis shows the normalized enrichment values. The adjusted *p* value and number of genes are also denoted by color and by size, respectively. **f** Venn diagram showing up- and downregulated DEGs in APP^V717I^ versus Ctrl neurons in grafts versus in vitro. **g** Pathway enrichment analysis of DEGs uniquely found in grafted APP^V717I^ neurons. The *x*-axis shows the normalized enrichment values. The adjusted *p* value and number of genes are also denoted by color and by size, respectively

Encouraged by the successful survival of neuronal grafts, we next asked if the grafted APP^V717I^ neurons carried altered gene expression signatures in the adult brain. We micro-dissected APP^V717I^ and Ctrl grafts from the brains and performed single nucleus RNA-sequencing (snRNA-seq), which captures both engrafted human cells and nearby host cells (Fig. 3a). Within the cluster of human nuclei from all grafts, we identified a total of 15,699 APP^V717I^ and 20,497 Ctrl nuclei, with ~ 90% of nuclei annotated as neurons (14,367 APP^V717I^ neurons and 17,928 Ctrl neurons) and 10% as astrocytes (1332 APP^V717I^ astrocytes and 2569 Ctrl astrocytes), which is consistent with our histological analysis (Fig. 3b). Notably, there was a separation within the neuronal cluster with glutamatergic and GABAergic neurons when comparing the APP^V717I^ and Ctrl groups in the UMAP plot (Fig. 3c; Figure S3a–c). We identified differentially expressed genes (DEGs) in APP^V717I^ neurons and investigated the KEGG pathways that were enriched in APP^V717I^ versus Ctrl neurons (Fig. 3d and e; Table S1). As with our bulk RNA-seq analysis, many synaptic function-related pathways were dysregulated in AD neurons including glutamatergic synapse, GABAergic synapse, long-term potentiation, calcium signaling, and long-term depression. In addition, pathways linked to mitophagy, MAPK signaling pathway, axon guidance and Alzheimer’s disease were also enriched in APP^V717I^ neurons (Fig. 3e; Table S1). A clear separation of transcriptome profiles was also observed in grafted APP^V717I^ astrocytes with an enrichment of many KEGG pathways including glutamatergic synapse, axon guidance and Rap and Ras signaling pathways, that play important roles in the pathogenesis of AD (Figure S4a–c; Table S1).

To examine how the brain environment affects DEG patterns in APP^V717I^ neurons, we compared differences in DEGs in human neuronal clusters comparing in vitro bulk-seq and in vivo pseudobulk seq data (Fig. 3f and g). We found that synaptic function-related pathways including glutamatergic synapse and long-term depression, calcium signaling pathways as well as neurodevelopment related pathways, including cell adhesion molecules and axon guidance, are further enriched in grafts compared to cultured cells. These results suggest that the in vivo environment promotes the development of synaptic function and the maturation of neurons, and they highlight synaptic dysfunction in APP^V717I^ neurons in vivo (Fig. 3f and g).

We next investigated gene expression profiles in host cells within and adjacent to APP^V717I^ and Ctrl grafts to interrogate non-cell autonomous transcriptomic changes induced by grafted human cells (Figure S2c–g). We identified the major murine cell types including neurons, microglia, astrocytes, oligodendrocytes, and endothelial cells (Figure S2c–d). Interestingly, the host neurons within and adjacent to APP^V717I^ grafts demonstrated significantly altered gene expression profiles (Figure S2c, d), with 65 upregulated and 142 downregulated DEGs in mouse neurons in the APP^V717I^ group (Figure S2e, f; Table S1). Pathway analysis of these DEGs revealed many perturbed cellular pathways that are essential for normal brain function, including several aforementioned synaptic function-related pathways (glutamatergic synapse, GABAergic synapse, long-term potentiation, calcium signaling, and long-term depression), small GTPase-mediated pathways, axon guidance, and the MAPK signaling pathway (Figure S2g; Table S1). These results indicate that engrafted human APP^V717I^ neurons and astrocytes induce significant cell-extrinsic changes in previously healthy neighboring murine host neurons.

### Comparative single nucleus RNA-seq analysis of grafted APP^V717I^ and human postmortem AD neurons captures overlapping transcriptome signatures

**Fig. 4:**
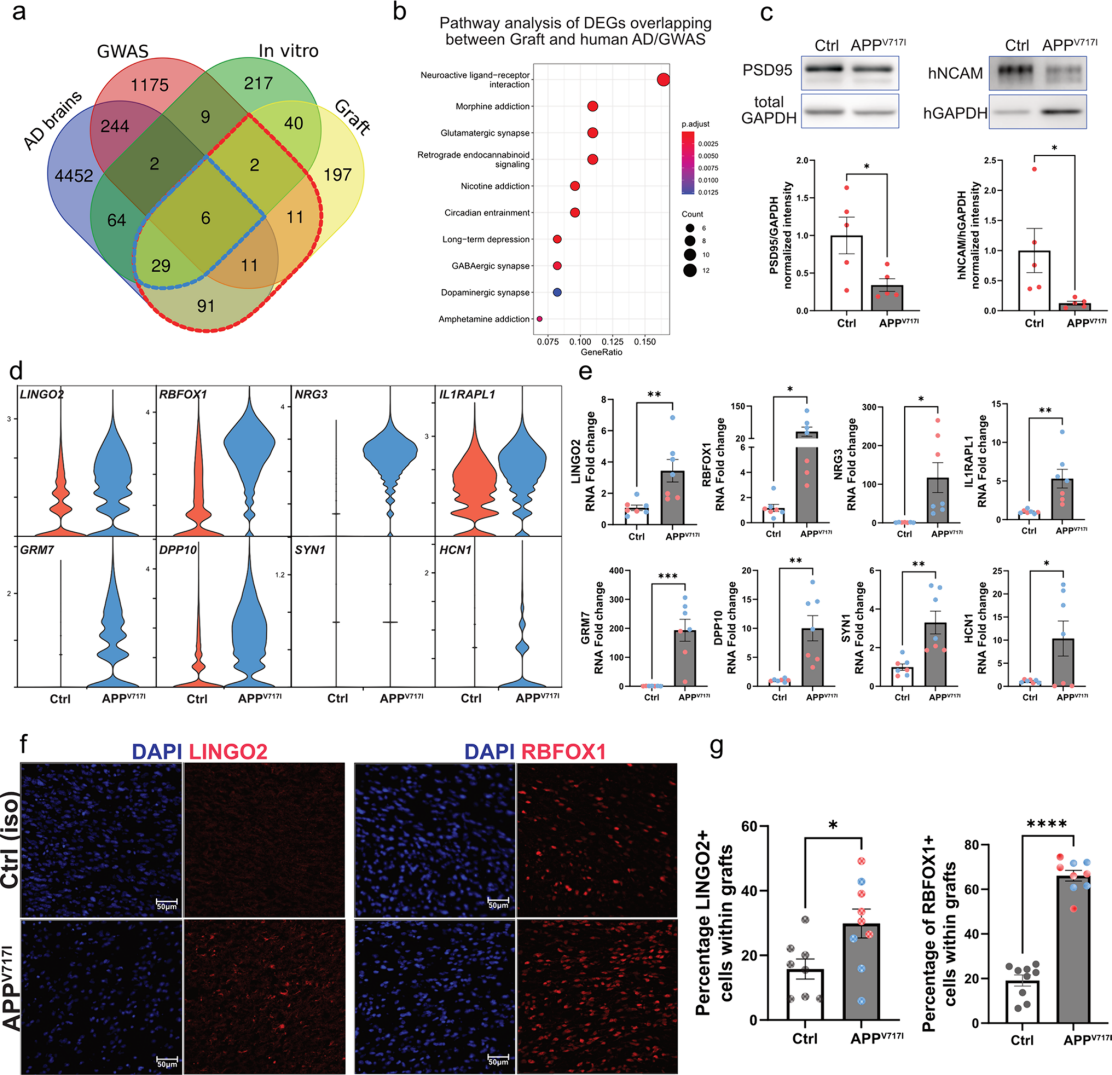
Comparative single nucleus RNA-seq analysis of grafted and human postmortem APP^V717I^ neurons captures overlapping transcriptome signatures. **a** Venn diagram of upregulated DEGs derived from snRNA-seq of human postmortem AD versus Ctrl neurons, AD GWAS hits (GWAS Catalog; EMBL-EBI), snRNA-seq of transplanted human APP^V717I^ versus Ctrl neurons, and cultured APP^V717I^ versus Ctrl neurons. Red circle indicates DEGs overlapping between grafted APP^V717I^ neurons and human AD brains/GWAS. Blue circle indicates DEGs overlapping between in vitro, graft and human AD brains/GWAS. **b** Pathway analysis of DEGs overlapping between all 4 groups (red circle in **a**). Grafted APP^V717I^ neurons show preservation of dysfunctional pathways of AD brains that are related to synaptic function. The *x*-axis shows the gene ratio. The adjusted *p* value and number of genes are also denoted by color and by size, respectively. **c** Western blot images with quantification after micro-dissection of APP^V717I^ and Ctrl grafts showing reduced expression of PSD95 and hNCAM in APP^V717I^ grafts. **d** Violin plots showing expression levels of indicated genes in grafted APP^V717I^ and Ctrl neurons. **e** RTqPCR validation of the expression of selected DEGs in cultured APP^V717I^ versus Ctrl neurons. **f** and **g** Immunostainings (**f**) with quantification (**g**) for the validation of increased expression of LINGO2 and RBFOX1 in grafted human APP^V717I^ cells. Student *t*-test was used in **c**, **e** and **g**, *p < 0.05, **p < 0.01, ***p < 0.001, ****p < 0.0001

To further characterize the single cell transcriptome profile in grafted APP^V717I^ neurons, we compared upregulated DEGs of in vitro-cultured and grafted APP^V717I^ neurons with published DEGs from cortical neurons profiled from postmortem AD brain tissue from individuals in the ROS and MAP cohorts [10, 34] (Fig. 4a). We also included an additional group of AD-associated GWAS hits [60] (https://www.ebi.ac.uk/gwas/) for this comparative analysis to expand the pool of AD-relevant genes and also to curate the list of overlapping genes with importance to AD (Fig. 4a). Notably, almost 40% of the upregulated DEGs in APP^V717I^ grafts are also found in human AD brains (compared to healthy controls) or in AD GWAS studies (150 out of 387 DEGs; Fig. 4a; red circle; Table S1). KEGG pathway analysis of these shared DEGs revealed changes in neuroactive ligand–receptor interaction, axon guidance and synaptic function-related pathways in APP^V717I^ neurons, including glutamatergic and GABAergic synapse (Fig. 4b; Table S1). Although there are substantial differences in maturity and circuit-formation between our grafted neurons and aged neurons from postmortem tissue, these results indicate that important AD-related transcriptional changes, in particular synapse-associated ones, can be captured in our chimeric transplantation model of AD. Immunoblotting of micro-dissected APP^V717I^ and Ctrl grafts further confirmed the phenotype of impaired neurite and synaptic health, as APP^V717I^ grafts showed a significant reduction of hNCAM and PSD95 proteins compared to Ctrl grafts (Fig. 4c).

To further validate this finding and to evaluate if these early transcriptional phenotypes in grafted APP^V717I^ neurons translate into changes in neuronal survival and neurite extension in the mouse brain later-on, we transplanted APP^V717I^ and Ctrl NPCs into the cortex and striatum of adult mice followed by histological analysis 12 months after cell injection (Figure S5). While all mice contained surviving grafts, the graft size and the total number of surviving neurons were significantly reduced in the APP^V717I^ group (Figure S5a-c). Amyloid plaque pathology was not observed in the grafts, but the APP^V717I^ grafts contained an increased number of p-tau+ (AT8+) neurons with diffuse cytoplasmic staining without tangle formation (Figure S5d-e). Concordantly, MC1 stainings for conformationally altered tau were negative (Figure S5f). A small number of cells in both groups (less than 1%) was positive for the apoptosis marker cleaved caspase 3 (Figure S5g) or for the phosphorylated form of the necroptosis marker Receptor Interacting Protein Kinase 1 (pRIP1) (Figure S5h). We then examined the outgrowth of transplanted APP^V717I^ and Ctrl neurons in the brains 12 months after injection and found pronounced impairment of neurite extension into the surrounding brain parenchyma with reduced number of hNCAM^+^ axonal projections in the cortex, basal ganglia, and white matter tracts of the ipsilateral and contralateral hemispheres in the APP^V717I^ group (Figure S5i-p).

We next focused on the DEGs that are consistently upregulated in cultured and 2 month-grafted APP^V717I^ neurons and in neurons from postmortem AD brain tissue (Fig. 4a blue circle; Fig. 4d, Figure S3d, Table S1). Based on the overall expression levels and expression fold-changes in AD neurons, their cellular function, and their relevance to AD, we selected 12 genes for validation by RTqPCR in cultured APP^V717I^ neurons. 8 of these genes (*LINGO2*, *RBFOX1*, *NRG3*, *IL1RAPL1*, *GRM7*, *DPP10*, *SYN1*, and *HCN1*) were significantly upregulated (Fig. 4e), while 4 of these genes (*GABRA2*, *PCDH11X*, *KHDRBS2*, and *PLCXD3*) demonstrated only a trend towards increased expression in the APP^V717I^ group (Figure S3e). Among these 8 significantly upregulated genes, *LINGO2* and *RBFOX1* are also listed in AD GWAS studies and thus, we further validated an upregulation of these two markers in APP^V717I^ grafts by immunostaining (Fig. 4f and g). In contrast to upregulated DEGs, only few genes (*TCEAL2*, *EBF1*, *EBF2*, *MAP1LC3A*, *ZNF429*, *ZNF793*, *ZNF248* and *ZNF680*) were commonly downregulated in cultured, grafted and postmortem AD neurons, all of which demonstrated relatively low expression levels in APP^V717I^ grafts (Figure S3f, g).

### Downregulation of LINGO2 rescues neurite outgrowth deficits and reverses gene expression of AD-related pathways in APP^V717I^ neurons

**Fig. 5:**
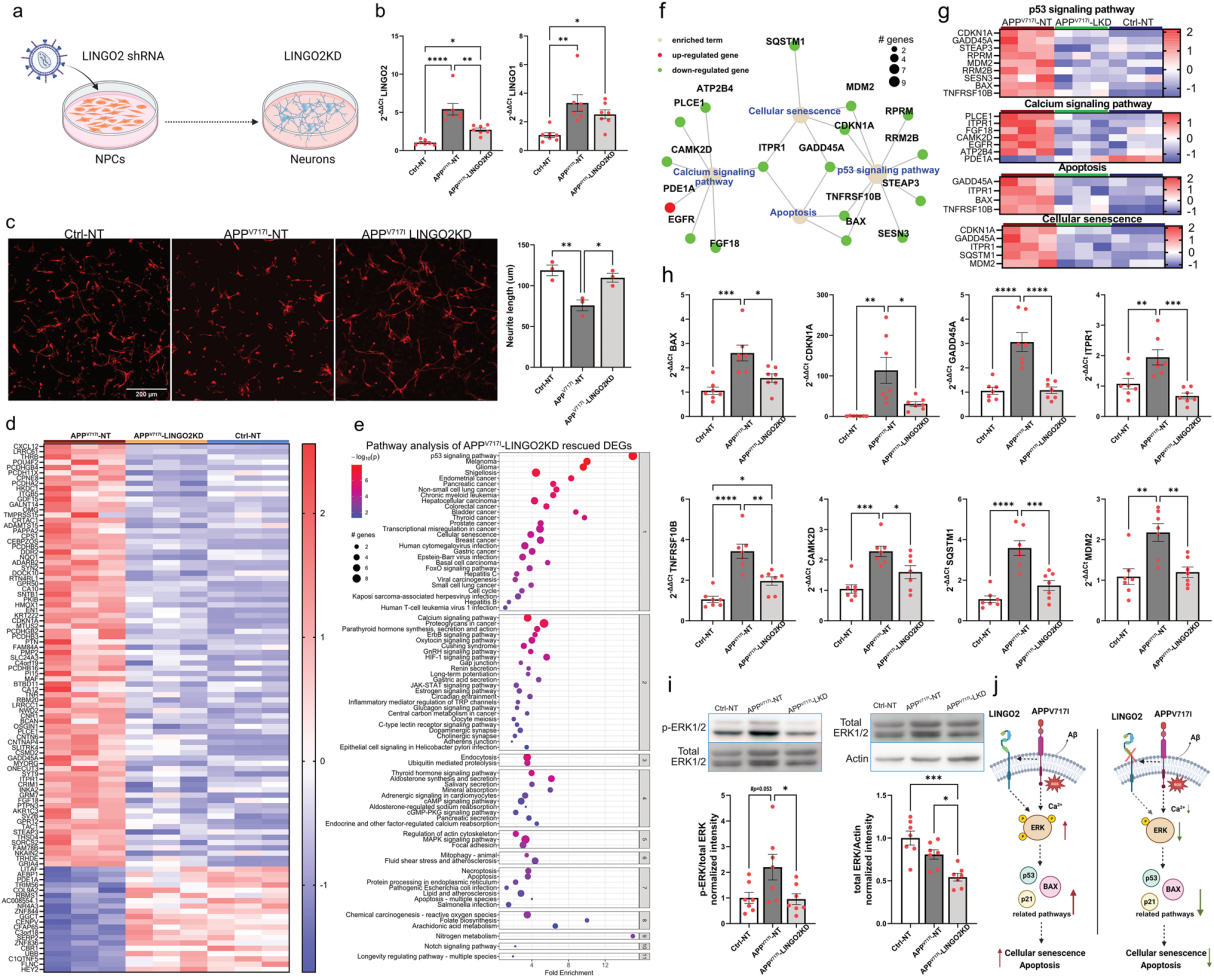
Downregulation of LINGO2 rescues neurite outgrowth deficits and reverses gene expression of AD-related pathways in APP^V717I^ neurons. **a** Schematic illustration of shRNA-mediated downregulation of LINGO2 in APP^V717I^ neurons in vitro*.* **b** RTqPCR demonstrates successful downregulation of LINGO2, but not LINGO1, in APP^V717I^ neurons. **c** Images and quantifications of neurite outgrowth show that LINGO2 knockdown (LINGO2KD) rescues the neurite outgrowth deficit of APP^V717I^ cells. **d** Heatmap showing the top 100 most variable rescued DEGs after LINGO2 knockdown in APP^V717I^ cells. **e** Pathway enrichment analysis of rescued DEGs by LINGO2 knockdown in APP^V717I^ neurons. The *x*-axis shows the normalized enrichment values. The adjusted *p* value and number of genes are also denoted by color and by size, respectively. **f** Term gene graph showing functional overlap between selected rescued pathways. **g** Heatmaps of rescued genes in selected pathways. **h** RTqPCR validation of selected key modulating genes. **i** Western blot images with quantification of the expression of p-ERK and ERK in Ctrl and APP^V717I^ neurons with and without LINGO2 knockdown. **j** Schematic drawing summarizing the downstream effects of LINGO2 downregulation in APP^V717I^ neurons. In APP^V717I^ neurons, LINGO2 expression is increased leading to hyperactivated ERK that results in increased signaling in p53, BAX, and p21-related pathways and contributes to cellar senescence and apoptosis. Downregulation of LINGO2 alleviates the hyperphosphorylation of ERK that attenuates cellular senescence and apoptosis driven by p53, BAX, and p21-related pathways. One-way ANOVA with Tukey post-hoc test was used in **b**, **c**, **h** and **i**, *p < 0.05, **p < 0.01, ***p < 0.001, ****p < 0.0001. Ctrl-NT and APP^V717I^-NT: Ctrl and APP^V717I^ neurons transduced with non-target-control virus

Having identified LINGO2 and RBFOX1 as key genes that show conserved changes in our in vitro system as well as in human genetic and postmortem tissue data, we asked if targeting LINGO2 and RBFOX1 would lead to a rescue of disease phenotypes in APP^V717I^ neurons, thereby establishing these genes as potential therapeutic targets in AD. While downregulation of RBFOX1 showed only a limited effect on the phenotypes of APP^V717I^ neurons (Figure S6a–g), LINGO2 showed more pronounced effects in our targeting experiments. LINGO2 (leucine rich repeat and Ig domain containing 2) is a transmembrane protein and a homolog of LINGO1, which negatively regulates neuronal growth and cell survival [27]. To assess LINGO2 function in APP^V717I^ neurons, LINGO2 knockdown NPC lines were generated by transducing NPCs with lentiviral particles containing LINGO2 shRNA (Fig. 5a). Downregulation of LINGO2 in differentiated APP^V717I^ neurons was confirmed by RTqPCR and did not affect the expression of LINGO1, which was also upregulated in AD neurons (Fig. 5b). We next performed neurite outgrowth assays by measuring lengths of neurons 24 h after replating at the end of differentiation [14]. These assays confirmed our previous findings of impaired neurite extension in APP^V717I^ neurons (Fig. 1e), with reduced neurite lengths in non-target-shRNA transduced APP^V717I^ neurons (AD-NT) compared to non-target-shRNA transduced Ctrl neurons (Ctrl-NT) (Fig. 5c). However, LINGO2 knockdown in APP^V717I^ neurons (APP^V717I^-LINGO2KD) showed a significant improvement of neurite outgrowth (Fig. 5c). To ensure that the neurite outgrowth changes were not influenced by a potential vulnerability caused by replating neurons, we also measured neurite lengths in these three groups after 5 days of differentiation and a similar rescue effect of LINGO2 knockdown on neurite outgrowth was observed in our APP^V717I^ neurons (Figure S7a-b). Together, these data suggest that LINGO2 knockdown rescued neurite outgrowth deficits of APP^V717I^ neurons.

We next performed bulk RNA-seq on differentiated Ctrl-NT, APP^V717I^-NT and APP^V717I^-LINGO2KD neurons to characterize genes and pathways that are rescued by LINGO2 knockdown (Fig. 5d–g; Table S1). Principal component analysis demonstrated that these three groups formed separate clusters (Figure S7c). Compared to APP^V717I^-NT neurons, 407 genes were significantly upregulated, and 520 genes were significantly downregulated in APP^V717I^-LINGO2KD neurons (Figure S7d; Table S1). We then selected genes from this DEG pool, whose expression is similar in Ctrl-NT and APP^V717I^-LINGO2KD neurons but significantly different in APP^V717I^-NT neurons. A total of 191 genes were identified and the top 100 most variable genes are shown in Fig. 5d (see also Table S1). Pathway analysis of these DEGs identified several AD-related pathways including the p53 signaling pathway, apoptosis, necroptosis, mitophagy, and cellular senescence and synapse-related pathways such as calcium signaling, which were rescued by LINGO2 KD (Fig. 5g-h; Table S1). Rescued expression of key DEGs in these pathways showed some functional overlap (Fig. 5f), and were validated by RTqPCR, including *BAX*, *ITPR1*, *CAMK2D*, *GADD45A*, *SQSTM1*, *TNFRSF10*, *MDM2*, and *CDKN1A* (Fig. 5h). Interestingly, LINGO2 KD increased Aβ_42_ production without significantly altering the Aβ_42_/Aβ_40_ ratio, and it had no effect on p-tau levels (Figure S7e-i), indicating that the rescue effects on aforementioned pathways may be independent of Aβ and tau pathology in APP^V717I^-LINGO2KD neurons.

The KEGG pathway analysis also identified alterations in the ERK/MAPK signaling pathway (Fig. 5e). The ERK signaling pathway has been associated with p53-mediated apoptosis and promotes cellular senescence [67, 80], and hyperphosphorylation of ERK has been linked to the pathogenesis of AD [32]. Thus, we performed immunoblotting for ERK and phospho-ERK on neuronal lysates and found a decrease of total ERK and p-ERK/ERK in APP^V717I^ neurons after LINGO2 KD (Fig. 5i). These findings indicate that downregulation of p-ERK in APP^V717I^ neurons via LINGO KD might at least in part explain observed beneficial effects on p53-, apoptosis-, and cellular senescence-associated pathways (Fig. 5j).

## Discussion

Here, we characterized pathological and transcriptomic changes in human iPSC-derived neurons carrying the *APP*^*V717I*^ mutation and uncovered previously unrecognized transcriptomic alterations induced by this common fAD mutation. In vitro differentiated APP^V717I^ neurons exhibited elevated levels of Aβ_42_ and p-tau, consistent with published literature demonstrating that the *APP*^*V717I*^ mutation alters APP cleavage, favors Aβ_42_ production and induces tau pathology [41, 48, 68]. In addition, we observed mitochondrial dysfunction, neurite outgrowth deficits, and altered polar metabolite profiling in APP^V717I^ neurons compared to their isogenic Ctrl in vitro*,* as similarly described in various amyloid models [7, 8, 39, 49, 63, 74]. Xenografted NPCs successfully survived and differentiated into neurons and astrocytes in adult mouse brains. snRNA-seq analysis of micro-dissected human grafts revealed significantly altered transcriptional profiles in APP^V717I^ neurons, including those related to dysregulated synaptic function. These changes were further highlighted by comparing DEGs of APP^V717I^ grafts with data from human postmortem AD brains and GWAS studies.

The transcriptome changes in grafted human APP^V717I^ neurons may be a result of both cell autonomous and non-cell autonomous effects that are mediated, at least in part, by Aβ. The *APP*^*V717I*^ mutation increases the production of Aβ_42_ in neurons, and there is increasing evidence that pre-fibrillar soluble oligomeric Aβ species play a central role in AD pathogenesis, damping neuronal function and contributing to brain damage in AD, that led to the Aβ oligomer (AβO) hypothesis [59, 72]. In support of this hypothesis, a single exposure to low levels of Aβ_42_ is sufficient to induce synapse-related gene expression changes in iPSC-derived neurons and calcium signaling alterations in iPSC-derived astrocytes that may contribute to early changes in neuronal network activities [36]. In addition, increased intracellular Aβ production can affect cellular pathways and synaptic health that precedes plaque formation [6, 17]. In line with these studies, the transcriptome changes we observed in grafted human APP^V717I^ neurons and astrocytes can be explained by the exposure to Aβ_42_ produced by neighboring APP^V717I^ cells and by APP cleavage alterations intracellularly. Such increased exposure to Aβ may also explain some of the synaptic functional changes in early AD brains that proceed plaques, tangles, and neuronal cell death [18].

The injection of human APP^V717I^ neurons and astrocytes resulted in significant transcriptional alterations in previously healthy neighboring murine host neurons, disrupting many cellular pathways essential for normal brain function, including those related to synaptic function. These effects are likely induced by Aβ_42_ secreted by human APP^V717I^ neurons, but other secreted factors or direct contact with APP^V717I^ cells may also contribute to the transcriptome changes. These findings further underscore the non-cell autonomous effects of the APP^V717I^ mutation on cellular function.

Our cell injection model complements previously published neuronal chimeric models related to AD, which have proven instrumental in AD research [28]. The injection of human embryonic stem cell (ESC)-derived NPCs into immunocompromised *APP/PS1-21*-NOD-SCID mice [15] or *Rag2*^*−/−*^*/App*^*NL−G−F*^ mice [5] resulted in successful engraftment of neurons exhibiting key AD pathological features in the presence of amyloid plaques, which were not seen after cell injection into NOD-SCID or *Rag2*^*−/−*^ control mice devoid of Aβ pathology. These pathological features included amyloid-associated neurite dystrophy 4 months after cell injection [15], formation of p-tau positive tangles in human neurons 18 months after cell injection, and increased death of human neurons linked to MEG3-associated necroptosis, which was noted already 6 months after cell injection [5]. Besides amyloid-induced toxicity, chimeric models have been employed to investigate AD risk factors, such as APOE4. The injection of human iPSC-derived APOE4/4 neurons into E4KI mice has revealed dysregulated pathways encompassing synaptic function, calcium homeostasis, and apoptosis 7 months after cell injection [42]. Complementing these studies, we have noted changes in AD-related synaptic gene expression signatures in xenografted APP^V717I^ neurons 2 months after cell injection, and we have identified neuronal cell loss, neurite outgrowth deficits and p-tau pathology in APP^V717I^ neurons 12 months post-injection. Interestingly, transplanted APP^V717I^ neurons did not induce plaque formation at both time points. The absence of plaque formation could be related to an intact amyloid clearing system in the host brains, to the still relatively limited observation time of up to 12 months post-injection, and to an elevated Aβ production that may not be sufficient to induce plaque formation in the mouse brains. Genetically modified amyloid mouse models often overexpress the human *APP* gene with multiple familial AD mutations but still require several months to develop plaques [55]. A heterozygous *APP* knock-in mouse model of AD that harbors the Swedish and Beyreuther/Iberian mutations (App^NL−F/wt^ mice) exhibits cortical amyloidosis only after 24 months [54]. Our human APP^V717I^ neurons, which are heterozygous for the fAD mutation, were xenografted in a small portion of the brains of NSG mice for up to 12 months. Consequently, a lack of plaque formation is not surprising. However, pronounced transcriptome alterations were evident in xenografted APP^V717I^ neurons despite the lack of plaque formation, highlighting a possible substantial toxicity of soluble Aβ_42_ oligomers. The lack of amyloid plaques may also explain observed differences in necroptotic cell death, which was low in our 12-month APP^V717I^ grafts but significantly induced by amyloid plaques in human ESC-derived neural grafts [5]. Despite variations in study strategies and the focus on different genetic variants, time points of analysis and mouse models for cell injection, the convergence of various disease phenotypes in our and published studies suggests a shared disturbance in several cellular pathways that may contribute to the pathogenesis of AD.

LINGO2 emerged as one of the upregulated genes in the neurons of both APP^V717I^ grafts and in postmortem brain tissue from donors with AD. LINGO2 is a paralog of LINGO1 with assumed similar function as LINGO1 [13], which increases neuronal vulnerability and inhibits neurite regeneration in a spinal cord lesion model [27]. LINGO1 and LINGO2 are associated with increased risk of essential tremor and Parkinson’s disease, and a single nucleotide polymorphism of LINGO2 is associated with increased risk of AD [43, 71]. LINGO1, along with LINGO2 and LINGO3, promotes lysosomal degradation of APP in immortalized cell lines of AD, leading to a decrease in Aβ production from the amyloidogenic pathway [13], while beneficial effects of LINGO1 blockage on amyloid burden have also been reported in AD mouse models [23]. We found that LINGO2 downregulation elevated Aβ_42_ levels in APP^V717I^ neurons but it is currently not known if LINGO2 interacts with APP, as described for LINGO1 that directly interacts with APP via its ectodomain [4, 65]. LINGO2 and LINGO1 proteins share 61% sequence similarity [38] encouraging future studies to precisely examine a potential interaction of LINGO2 with wildtype and mutant forms of APP. Notably, a recent study revealed that cell-secreted Aβ upregulates genes related to synaptic function, with LINGO2 identified among DEGs in human induced neurons [36]. This finding suggests that LINGO2 expression levels can be elevated by increased Aβ secretion, which triggers signaling cascades that may contribute to cellular damage.

Consistent with reported functions of LINGO1/LINGO2, downregulation of LINGO2 rescued neurite outgrowth deficits in APP^V717I^ neurons. In addition, the LINGO2 KD reversed several transcriptome signatures in APP^V717I^ neurons to Ctrl levels, including those related to synaptic function as well as apoptosis and cellular senescence, which are activated in AD in addition to alternative cell death-associated pathways [24, 70]. Recent work demonstrated that AD patient brains contain a significantly higher number of neurons that express senescence marker, and cultured AD neurons (iNs) displayed a senescence-like state with a senescence-associated pro-inflammatory phenotype [24]. Regulation of senescence and cell viability has been linked to the ERK signaling pathway [9, 58, 76]. While the effects of ERK signaling depend on cell type, duration and magnitude of activation [9, 66], its activation can be pro-apoptotic and promotes cellular senescence via p53 and p21 pathways [31, 32, 67]. Furthermore, its activation supports cell death in cultured neurons [66]. Here, we demonstrate an enrichment of the ERK/MAPK signaling pathway in APP^V717I^ neurons, which was reversed after LINGO2 KD. In addition, downregulation of LINGO2 reduced the levels of p-ERK and total ERK in APP^V717I^ neurons. These findings are consistent with previous reports that showed reduced phosphorylation of ERK after LINGO2 downregulation in cancer cells [31]. Thus, our findings suggest that LINGO2 KD confers beneficial effects in APP^V717I^ neurons by affecting cellular signaling pathways including ERK/MAPK signaling with a downregulation of apoptosis- and senescence-associated genes. Future studies examining the effect of LINGO2 KD in vivo and evaluating its effect in combination with anti-amyloid drugs might provide additional insights into LINGO2 as a potential therapeutic target in AD.

Our transplantation approach could be applied to study the effects of selected compounds of interest on APP^V717I^ neurons and their microenvironment in vivo and to assess their blood brain barrier penetrance and efficacy in preclinical settings. Yet, despite its strong translational value, there are some limitations to this study. First, only male mice and only female APP^V717I^ and Ctrl cells were used in this study, while sex-differences in astroglial and microglial reactivity [1, 19] and in AD pathology [46] have been described. This is partially mitigated by our comparison with published data from postmortem human tissue that included balanced sampling of donors of both sexes. Future comparisons between female and male mice as well as female and male cells for injection could identify sex-specific changes in AD disease phenotypes. Second, APP^V717I^ and Ctrl cells were injected into the forebrain of NSG mice lacking amyloid or tau pathology. Since AD pathology in host brains can accelerate and aggravate pathology in transplanted cells [5, 15], future comparative studies could include such models as well as different sites of injection and time points of analysis to assess additional cell-extrinsic effects of the host microenvironment on grafted APP^V717I^ neurons. Third, there are many differences between the DEGs in human postmortem AD neurons and the DEGs in grafted APP^V717I^ neurons. These differences could be related to the mutational status of neurons and to the presence of AD histopathological changes in human brains but not in grafts including amyloid plaques, neurofibrillary tangles and reactive microglia. Fourth, we downregulated LINGO2 in APP^V717I^ neurons and tested rescue of neurite outgrowth and transcriptional dysregulation only in vitro since those phenotypes were very prominent in cultured neurons. Future studies could test the effects of LINGO KD in transplanted APP^V717I^ neurons, which might provide additional information about the function of LINGO2 on neuronal health. In addition, it is very unlikely that LINGO2 is the only driver of AD pathogenesis. Several other potential therapeutic targets surfaced with our approach and their disease mechanisms remain to be further explored.

### Supplementary Information

Below is the link to the electronic supplementary material.

Supplementary file1 (PDF 176 kb)

Supplementary file2 (PDF 13287 kb)

Supplementary file3 (XLSX 16632 kb)

Supplementary file4 (XlSX 4045 kb)

## References

[1] Acaz-Fonseca E, Duran JC, Carrero P, Garcia-Segura LM, Arevalo MA (2015). Sex differences in glia reactivity after cortical brain injury. Glia.

[2] Al-Dalahmah O, Lam M, McInvale JJ, Qu W, Nguyen T, Mun JY (2024). Osteopontin drives neuroinflammation and cell loss in MAPT-N279K frontotemporal dementia patient neurons. Cell Stem Cell.

[3] Al-Dalahmah O, Sosunov AA, Shaik A, Ofori K, Liu Y, Vonsattel JP (2020). Single-nucleus RNA-seq identifies Huntington disease astrocyte states. Acta Neuropathol Commun.

[4] Bai Y, Markham K, Chen F, Weerasekera R, Watts J, Horne P (2008). The in vivo brain interactome of the amyloid precursor protein. Mol Cell Proteom.

[5] Balusu S, Horre K, Thrupp N, Craessaerts K, Snellinx A, Serneels L (2023). MEG3 activates necroptosis in human neuron xenografts modeling Alzheimer's disease. Science.

[6] Bayer TA, Wirths O (2010). Intracellular accumulation of amyloid-beta—a predictor for synaptic dysfunction and neuron loss in Alzheimer's disease. Front Aging Neurosci.

[7] Bhatia S, Rawal R, Sharma P, Singh T, Singh M, Singh V (2022). Mitochondrial dysfunction in Alzheimer's disease: opportunities for drug development. Curr Neuropharmacol.

[8] Blazquez-Llorca L, Valero-Freitag S, Rodrigues EF, Merchan-Perez A, Rodriguez JR, Dorostkar MM (2017). High plasticity of axonal pathology in Alzheimer's disease mouse models. Acta Neuropathol Commun.

[9] Cagnol S, Chambard JC (2010). ERK and cell death: mechanisms of ERK-induced cell death–apoptosis, autophagy and senescence. FEBS J.

[10] Cain A, Taga M, McCabe C, Green GS, Hekselman I, White CC (2023). Multicellular communities are perturbed in the aging human brain and Alzheimer's disease. Nat Neurosci.

[11] Caldwell AB, Liu Q, Schroth GP, Galasko DR, Yuan SH, Wagner SL (2020). Dedifferentiation and neuronal repression define familial Alzheimer's disease. Sci Adv.

[12] Chow VW, Mattson MP, Wong PC, Gleichmann M (2010). An overview of APP processing enzymes and products. Neuromol Med.

[13] de Laat R, Meabon JS, Wiley JC, Hudson MP, Montine TJ, Bothwell M (2015). LINGO-1 promotes lysosomal degradation of amyloid-β protein precursor. Pathobiol Aging Age Relat Dis.

[14] Ehrlich M, Hallmann AL, Reinhardt P, Arauzo-Bravo MJ, Korr S, Ropke A (2015). Distinct neurodegenerative changes in an induced pluripotent stem cell model of frontotemporal dementia linked to mutant TAU protein. Stem Cell Rep.

[15] Espuny-Camacho I, Arranz AM, Fiers M, Snellinx A, Ando K, Munck S (2017). Hallmarks of Alzheimer's disease in stem-cell-derived human neurons transplanted into mouse brain. Neuron.

[16] Espuny-Camacho I, Michelsen KA, Gall D, Linaro D, Hasche A, Bonnefont J (2013). Pyramidal neurons derived from human pluripotent stem cells integrate efficiently into mouse brain circuits in vivo. Neuron.

[17] Gallego Villarejo L, Bachmann L, Marks D, Brachthauser M, Geidies A, Muller T (2022). Role of intracellular amyloid beta as pathway modulator, biomarker, and therapy target. Int J Mol Sci.

[18] Gazestani V, Kamath T, Nadaf NM, Dougalis A, Burris SJ, Rooney B (2023). Early Alzheimer's disease pathology in human cortex involves transient cell states. Cell.

[19] Guneykaya D, Ivanov A, Hernandez DP, Haage V, Wojtas B, Meyer N (2018). Transcriptional and translational differences of microglia from male and female brains. Cell Rep.

[20] Hallmann AL, Arauzo-Bravo MJ, Mavrommatis L, Ehrlich M, Ropke A, Brockhaus J (2017). Astrocyte pathology in a human neural stem cell model of frontotemporal dementia caused by mutant TAU protein. Sci Rep.

[21] Hargus G, Cooper O, Deleidi M, Levy A, Lee K, Marlow E (2010). Differentiated Parkinson patient-derived induced pluripotent stem cells grow in the adult rodent brain and reduce motor asymmetry in Parkinsonian rats. Proc Natl Acad Sci USA.

[22] Hargus G, Ehrlich M, Arauzo-Bravo MJ, Hemmer K, Hallmann AL, Reinhardt P (2014). Origin-dependent neural cell identities in differentiated human iPSCs in vitro and after transplantation into the mouse brain. Cell Rep.

[23] He Q, Jiang L, Zhang Y, Yang H, Zhou C-N, Xie Y-H (2021). Anti-LINGO-1 antibody ameliorates cognitive impairment, promotes adult hippocampal neurogenesis, and increases the abundance of CB1R-rich CCK-GABAergic interneurons in AD mice. Neurobiol Dis.

[24] Herdy JR, Traxler L, Agarwal RK, Karbacher L, Schlachetzki JCM, Boehnke L (2022). Increased post-mitotic senescence in aged human neurons is a pathological feature of Alzheimer's disease. Cell Stem Cell.

[25] Holtzman DM, Morris JC, Goate AM (2011). Alzheimer's disease: the challenge of the second century. Sci Transl Med.

[26] Hu K, Yu J, Suknuntha K, Tian S, Montgomery K, Choi K-D (2011). Efficient generation of transgene-free induced pluripotent stem cells from normal and neoplastic bone marrow and cord blood mononuclear cells. Blood J Am Soc Hematol.

[27] Huang L-J, Li G, Ding Y, Sun J-H, Wu T-T, Zhao W (2019). LINGO-1 deficiency promotes nerve regeneration through reduction of cell apoptosis, inflammation, and glial scar after spinal cord injury in mice. Exp Neurol.

[28] Ifediora N, Canoll P, Hargus G (2024). Human stem cell transplantation models of Alzheimer's disease. Front Aging Neurosci.

[29] Israel MA, Yuan SH, Bardy C, Reyna SM, Mu Y, Herrera C (2012). Probing sporadic and familial Alzheimer's disease using induced pluripotent stem cells. Nature.

[30] Jerng HH, Qian Y, Pfaffinger PJ (2004). Modulation of Kv4. 2 channel expression and gating by dipeptidyl peptidase 10 (DPP10). Biophys J.

[31] Jo JH, Park SB, Park S, Lee HS, Kim C, Jung DE (2019). Novel gastric cancer stem cell-related marker LINGO2 is associated with cancer cell phenotype and patient outcome. Int J Mol Sci.

[32] Khezri MR, Yousefi K, Esmaeili A, Ghasemnejad-Berenji M (2023). The role of ERK1/2 pathway in the pathophysiology of Alzheimer's disease: an overview and update on new developments. Cell Mol Neurobiol.

[33] Malloy C, Ahern M, Lin L, Hoffman DA (2022). Neuronal roles of the multifunctional protein dipeptidyl peptidase-like 6 (DPP6). Int J Mol Sci.

[34] Mathys H, Davila-Velderrain J, Peng Z, Gao F, Mohammadi S, Young JZ (2019). Single-cell transcriptomic analysis of Alzheimer’s disease. Nature.

[35] McInvale JJ, Canoll P, Hargus G (2024). Induced pluripotent stem cell models as a tool to investigate and test fluid biomarkers in Alzheimer's disease and frontotemporal dementia. Brain Pathol.

[36] Melo de Farias AR, Pelletier A, Iohan LCC, Saha O, Bonnefond A, Amouyel P (2023). Amyloid-beta peptides trigger premature functional and gene expression alterations in human-induced neurons. Biomedicines.

[37] Mertens J, Herdy JR, Traxler L, Schafer ST, Schlachetzki JCM, Bohnke L (2021). Age-dependent instability of mature neuronal fate in induced neurons from Alzheimer's patients. Cell Stem Cell.

[38] Mi S, Lee X, Shao Z, Thill G, Ji B, Relton J (2004). LINGO-1 is a component of the Nogo-66 receptor/p75 signaling complex. Nat Neurosci.

[39] Milward EA, Papadopoulos R, Fuller SJ, Moir RD, Small D, Beyreuther K (1992). The amyloid protein precursor of Alzheimer's disease is a mediator of the effects of nerve growth factor on neurite outgrowth. Neuron.

[40] Misrani A, Tabassum S, Yang L (2021). Mitochondrial dysfunction and oxidative stress in Alzheimer’s disease. Front Aging Neurosci.

[41] Muratore CR, Rice HC, Srikanth P, Callahan DG, Shin T, Benjamin LN (2014). The familial Alzheimer's disease APPV717I mutation alters APP processing and Tau expression in iPSC-derived neurons. Hum Mol Genet.

[42] Najm R, Zalocusky KA, Zilberter M, Yoon SY, Hao Y, Koutsodendris N (2020). In vivo chimeric Alzheimer's disease modeling of apolipoprotein E4 toxicity in human neurons. Cell Rep.

[43] Nazarian A, Arbeev KG, Yashkin AP, Kulminski AM (2019). Genetic heterogeneity of Alzheimer’s disease in subjects with and without hypertension. Geroscience.

[44] O'Brien RJ, Wong PC (2011). Amyloid precursor protein processing and Alzheimer's disease. Annu Rev Neurosci.

[45] Ortiz-Virumbrales M, Moreno CL, Kruglikov I, Marazuela P, Sproul A, Jacob S (2017). CRISPR/Cas9-correctable mutation-related molecular and physiological phenotypes in iPSC-derived Alzheimer’s PSEN2 N141I neurons. Acta Neuropathol Commun.

[46] Oveisgharan S, Arvanitakis Z, Yu L, Farfel J, Schneider JA, Bennett DA (2018). Sex differences in Alzheimer’s disease and common neuropathologies of aging. Acta Neuropathol.

[47] Paglia G, Stocchero M, Cacciatore S, Lai S, Angel P, Alam MT (2016). Unbiased metabolomic investigation of Alzheimer’s disease brain points to dysregulation of mitochondrial aspartate metabolism. J Proteome Res.

[48] Peris L, Parato J, Qu X, Soleilhac JM, Lante F, Kumar A (2022). Tubulin tyrosination regulates synaptic function and is disrupted in Alzheimer's disease. Brain.

[49] Petratos S, Li QX, George AJ, Hou X, Kerr ML, Unabia SE (2008). The beta-amyloid protein of Alzheimer's disease increases neuronal CRMP-2 phosphorylation by a Rho-GTP mechanism. Brain.

[50] Qu W, Canoll P, Hargus G (2022). Molecular insights into cell type-specific roles in alzheimer’s disease: human induced pluripotent stem cell-based disease modeling. Neuroscience.

[51] Qu W, Jeong A, Zhong R, Thieschafer JS, Gram A, Li L (2023). Deletion of small GTPase H-Ras rescues memory deficits and reduces amyloid plaque-associated dendritic spine loss in transgenic Alzheimer’s mice. Mol Neurobiol.

[52] Reinhardt P, Glatza M, Hemmer K, Tsytsyura Y, Thiel CS, Hoing S (2013). Derivation and expansion using only small molecules of human neural progenitors for neurodegenerative disease modeling. PLoS ONE.

[53] Reinhardt P, Schmid B, Burbulla LF, Schondorf DC, Wagner L, Glatza M (2013). Genetic correction of a LRRK2 mutation in human iPSCs links parkinsonian neurodegeneration to ERK-dependent changes in gene expression. Cell Stem Cell.

[54] Saito T, Matsuba Y, Mihira N, Takano J, Nilsson P, Itohara S (2014). Single App knock-in mouse models of Alzheimer's disease. Nat Neurosci.

[55] Sasaguri H, Nilsson P, Hashimoto S, Nagata K, Saito T, De Strooper B (2017). APP mouse models for Alzheimer's disease preclinical studies. EMBO J.

[56] Sengoku R (2020). Aging and Alzheimer's disease pathology. Neuropathology.

[57] Serrano-Pozo A, Frosch MP, Masliah E, Hyman BT (2011). Neuropathological alterations in Alzheimer disease. Cold Spring Harb Perspect Med.

[58] Sipieter F, Cappe B, Leray A, De Schutter E, Bridelance J, Hulpiau P (2021). Characteristic ERK1/2 signaling dynamics distinguishes necroptosis from apoptosis. iScience.

[59] Sivanesan S, Tan A, Rajadas J (2013). Pathogenesis of Abeta oligomers in synaptic failure. Curr Alzheimer Res.

[60] Sollis E, Mosaku A, Abid A, Buniello A, Cerezo M, Gil L (2023). The NHGRI-EBI GWAS catalog: knowledgebase and deposition resource. Nucleic Acids Res.

[61] Song B, Cha Y, Ko S, Jeon J, Lee N, Seo H (2020). Human autologous iPSC-derived dopaminergic progenitors restore motor function in Parkinson's disease models. J Clin Investig.

[62] Song J-M, Kang M, Park D-H, Park S, Lee S, Suh YH (2021). Pathogenic GRM7 mutations associated with neurodevelopmental disorders impair axon outgrowth and presynaptic terminal development. J Neurosci.

[63] Sonntag KC, Ryu WI, Amirault KM, Healy RA, Siegel AJ, McPhie DL (2017). Late-onset Alzheimer's disease is associated with inherent changes in bioenergetics profiles. Sci Rep.

[64] Sproul AA, Jacob S, Pre D, Kim SH, Nestor MW, Navarro-Sobrino M (2014). Characterization and molecular profiling of PSEN1 familial Alzheimer's disease iPSC-derived neural progenitors. PLoS ONE.

[65] Stein T, Walmsley AR (2012). The leucine-rich repeats of LINGO-1 are not required for self-interaction or interaction with the amyloid precursor protein. Neurosci Lett.

[66] Subramaniam S, Unsicker K (2010). ERK and cell death: ERK1/2 in neuronal death. FEBS J.

[67] Sugiura R, Satoh R, Takasaki T (2021). ERK: a double-edged sword in cancer. ERK-dependent apoptosis as a potential therapeutic strategy for cancer. Cells.

[68] Sun J, Carlson-Stevermer J, Das U, Shen M, Delenclos M, Snead AM (2019). CRISPR/Cas9 editing of APP C-terminus attenuates β-cleavage and promotes α-cleavage. Nat Commun.

[69] Syed P, Durisic N, Harvey RJ, Sah P, Lynch JW (2020). Effects of GABAA receptor α3 subunit epilepsy mutations on inhibitory synaptic signaling. Front Mol Neurosci.

[70] Thal DR, Gawor K, Moonen S (2024). Regulated cell death and its role in Alzheimer's disease and amyotrophic lateral sclerosis. Acta Neuropathol.

[71] Vilariño-Güell C, Wider C, Ross OA, Jasinska-Myga B, Kachergus J, Cobb SA (2010). LINGO1 and LINGO2 variants are associated with essential tremor and Parkinson disease. Neurogenetics.

[72] Viola KL, Klein WL (2015). Amyloid beta oligomers in Alzheimer's disease pathogenesis, treatment, and diagnosis. Acta Neuropathol.

[73] Vuong CK, Wei W, Lee J-A, Lin C-H, Damianov A, De la Torre-Ubieta L (2018). Rbfox1 regulates synaptic transmission through the inhibitory neuron-specific vSNARE Vamp1. Neuron.

[74] Wilkins HM, Swerdlow RH (2017). Amyloid precursor protein processing and bioenergetics. Brain Res Bull.

[75] Wollmer MA (2010). Cholesterol-related genes in Alzheimer's disease. Biochim Biophys Acta.

[76] Yagoda N, von Rechenberg M, Zaganjor E, Bauer AJ, Yang WS, Fridman DJ (2007). RAS-RAF-MEK-dependent oxidative cell death involving voltage-dependent anion channels. Nature.

[77] Yang J, Zhao H, Ma Y, Shi G, Song J, Tang Y (2017). Early pathogenic event of Alzheimer’s disease documented in iPSCs from patients with PSEN1 mutations. Oncotarget.

[78] Yu J, Hu K, Smuga-Otto K, Tian S, Stewart R, Slukvin II (2009). Human induced pluripotent stem cells free of vector and transgene sequences. Science.

[79] Yu J, Vodyanik MA, Smuga-Otto K, Antosiewicz-Bourget J, Frane JL, Tian S (2007). Induced pluripotent stem cell lines derived from human somatic cells. Science.

[80] Zou J, Lei T, Guo P, Yu J, Xu Q, Luo Y (2019). Mechanisms shaping the role of ERK1/2 in cellular senescence (review). Mol Med Rep.

