# Supplementary material for: Xenografted human iPSC-derived neurons with the familial Alzheimer’s disease APPV717I mutation reveal dysregulated transcriptome signatures linked to synaptic function and implicate LINGO2 as a disease signaling mediator
Source: Acta Neuropathol. 2024 Jun 25;147(1):107. doi: 10.1007/s00401-024-02755-5 (PMC11199265; doi:10.1007/s00401-024-02755-5)
Supplement: Supplementary file 1 — Supplementary file1 (PDF 176 kb) [file 401_2024_2755_MOESM1_ESM.pdf]

Supplemental Material

Xenografted human iPSC-derived neurons with the familial Alzheimer's disease *APP*^{V717I} mutation reveal dysregulated transcriptome signatures linked to synaptic function and implicate LINGO2 as a disease signaling mediator

Wenhui Qu, Matti Lam, Julie J. McInvale, Jason A. Mares, Sam Kwon, Nelson Humala, Aayushi Mahajan, Trang Nguyen, Kelly A. Jakubiak, Jeong-Yeon Mun, Thomas G. Tedesco, Osama Al-Dalahmah, Syed A. Hussaini, Andrew A. Sproul, Markus D. Siegelin, Philip L. De Jager, Peter Canoll, Vilas Menon, and Gunnar Hargus

Materials and Methods

Protein isolation and immunoblotting

Cells were harvested in RIPA extraction buffer (Thermo Fisher, PI89901) and protein concentration was measured using the DC protein assay kit (Bio-Rad, 5000112) according to the manufacturer's instructions. Immunoblotting was conducted as previously described [12, 13]. Isolated total proteins were separated by 12% sodium dodecyl sulfate-polyacrylamide gel electrophoresis (SDS-PAGE) and transferred to PVDF membranes. The membranes were blocked in 5% milk then incubated in primary antibody solution overnight. On the following day, membranes were washed and incubated in corresponding HRP-conjugated secondary antibodies for 1h then imaged using the Bio-Rad ChemiDoc imaging system. Images were quantified using the ImageJ software. The following primary antibodies were used in this study: PHF1 (Gift from Dr. Peter Davies; 1:1000), PSD95 (Cell Signaling; 3450; 1:1000), β-Actin (Millipore Sigma; A5441; 1:3000), AT8 (Thermo Fisher; MN1020; 1:1000), p-Tau356 (Thermo Fisher; 44-751G; 1:1000); AT180 (Thermo Fisher; MN1040, 1:1000), p-Tau217 (Thermo Fisher; 44-744; 1:1000); 6E10 (BioLegend; 803001; 1:1000), HT7 (Thermo Fisher; MN1000; 1:700), LINGO2 (R&D; MAB36791; 1:300).

ELISA

The ELISA assay on cell lysates in Figure 1 was done using V-PLEX Plus Aβ Peptide Panel 1 (6E10) Kit (MSD, K15200G-1) following the manufacturer's instructions. The ELISA assays in LINGO2KD and RBFOX1KD cell lysates in Figure 5, Figure S6 and Figure S7 were performed using Amyloid beta 42 Human ELISA Kit, (Ultrasensitive: Invitrogen; KHB3544; or regular: Invitrogen; KHB3441) and Amyloid beta 40 Human ELISA Kit (Invitrogen; KHB3481) following the manufacturer's instructions.

Immunocytochemistry and quantification of cells

Cells were fixed for 20 minutes at room temperature with 4% paraformaldehyde (Electron Microscopy Sciences) in PBS then washed three times with PBS. Cells were then incubated in NH_4Cl (100mM in PBS) for 10 min to quench free aldehyde groups followed by 3 times of washes, 30 min blocking in 10% normal goat serum in PBS and primary antibody incubation in blocking buffer overnight at 4 °C. Next day, cells were washed three times with PBS, followed by incubation of secondary antibody in PBS for one hour. Cells were then mounted in mounting medium with DAPI (Vector, NC9029229). Cells were imaged with THUNDER imager microscope (Leica) and quantified with QuPath software detection method as previously described [1, 5]. Primary antibodies include TUBB3 (TUJ1; BioLegend; 802001; 1:500), GABA (Millipore Sigma; A2052; 1:500), and VGluT1 (abcam; ab227805; 1:500).

Seahorse mitochondrial stress assay

Oxygen consumption rate (OCR) was measured using a Seahorse XFe24 Analyzer (Agilent) as previously described [9]. Briefly, neurons were grown in maturation medium for 2-3 weeks till 80-90% confluency and were then placed in a CO_2-free incubator for 1h. Mitochondrial stress assay (Agilent, 103015-100) was performed in the Seahorse XF base medium (Agilent, 102353-100) containing 10 mM glucose, 2 mM glutamine, 1 mM pyruvate. 2 μM oligomycin (OM), 2 μM Carbonyl cyanide-4 (trifluoromethoxy) phenylhydrazone (FCCP), and 0.5 μM rotenone/antimycin (R/A) were then injected into each well in a sequential order. After the assay, cells were washed and freeze-dried overnight. The total DNA was then measured by CyQUANT kit (C7026) or Nanodrop Spectrophotometer (ThermoFisher). The OCR was normalized to total DNA amount that represents the cell density of each well.

Polar metabolite profiling

Polar metabolite profiling was conducted as previously described [4, 9, 10]. Briefly, after 4 weeks of neuronal differentiation, cells were lysed in 80% cold HPLC grade methanol and cell lysates were centrifuged at 14,000 RCF for 20 min at 4°C. Supernatants containing polar metabolites were profiled by mass spectroscopy at the Weill Cornell Medicine core facility [4]. The peak area for targeted metabolites were quantified and normalized to the concentration of proteins extracted from the remaining pellets. The pellets were treated 0.2M NaOH and heated for 20 min at 95°C with frequent vortex, followed by 50 min of 10,000 RPM centrifugation. The protein concentration of the supernatant was measured by DC protein assay (Bio-Rad). The metabolite data analysis was conducted using MetaboAnalyst online tools [11].

Lentiviral transduction of NPCs

Predesigned MISSION® shRNA Lentivirus were purchased from MilliporeSigma. Multiple clones were tested and TRCN0000164140 showed strong efficacy in knocking down LINGO2 *in vitro*. Backbone of pLKO.1-puro was chosen, and transduction was conducted as suggested by the manufacturer. NPCs were seeded at 50,000 cells/ml density and an MOI of 5 was used for transduction. MISSION® pLKO.1-puro Non-Target shRNA Control Transduction Particles (SHC016V; Sigma) were used for negative controls. For eGFP and mCherry labeling, lentiviral particles from VectorBuilder were used (LVM(VB900083-7716grp)-C and LVM(VB900084-0158zxv)-C, respectively).

Neurite outgrowth measurement

NPCs were cultured in neuronal induction media for 5 days and phase contrast images of live cells were taken at 20x using the THUNDER imager microscope (Leica). Alternatively, GFP- or

RFP labeled cells were used and were fixed at the same time point. In independent experiments, neurons were treated with Accutase at the end of differentiation (4 weeks) and cells were replated onto Matrigel-coated cover slips, followed fixation and TUJ1-staining 24 hours after plating, as previously described [3]. Only neurons with distinguishable neurites were imaged and quantified. At least 15 cells from six random fields were included for each differentiation and averaged as the neurite length for one biological replicate. Neurite length was measured using the ImageJ software.

RNA extraction and quantitative RT-PCR (RT-qPCR)

Total RNA from cells was extracted and purified using the RNeasy Mini kit (QIAGEN) following the instructions from the manufacturer. RNA concentration was measured using Nanodrop Spectrophotometer (ThermoFisher). RNA was then reverse-transcribed to cDNA using the First-Strand-cDNA-Synthesis kit (Origene NP100042). RT-qPCR was performed using Power SYBR Green PCR Master Mix (Applied Biosystems) and the QuantStudio Real-time PCR system (QuantStudio5, ThermoFisher). The following primers were used:

*LINGO2*_for 5'-GTGTCTCAGAACCTGCTGGAAAC-3', *LINGO2*_rev 5'-TCGCTGCAAGATCCAGAGAAGG-3';

*LINGO1*_for 5'-CTCCATGTTGCATGAGCTGCTC-3', *LINGO1*_rev 5'-GATTCCTCCAGTGTGGTCAGCT-3';

*IL1RAPL1*_for 5'-CTCTGTGGAAGAAGGTGACTTGG-3', *IL1RAPL1*_rev 5'-CTCCAGCAAGTTCCACTGTGTAC-3';

*RBFOX1*_for 5'-GTATCAAGAGCCTGTGTATGGCA-3', *RBFOX1*_rev 5'-TCGGCAGCATAAACTCGTCCGT-3';

*NRG3*_for 5'-TGCCGAGACAAGGACCTTGCAT-3', *NRG3*_rev 5'-TCACAACGGACTCCTTGGTAGC-3';

*GRM7*_for 5'-CCAGACCACAAACACCAGCAAC-3', *GRM7*_rev 5'-CCTGGCTTACATGGTAGTGTGC-3'

*DPP10*_for 5'-GGACCGAAACCAGTATGCTCTTC-3', *DPP10*_rev 5'-CTGGAATCCACTTCCTCTGCCA-3';

*SYN1*_for 5'-CGATGCCAAATATGACGTGCGTG-3, *SYN1*_rev 5'-AGCATCGCAGAGCCAGTATTGG-3';

*HCN1*_for 5'-ACGAGAAGGAGCCGTGGGTAAA-3', *HCN1*_rev 5'-ACGTCCTTTGGTCAGCAGGCAA-3';

*GABRA2*_for 5'-CCCAATGCACTTGGAGGATTTCC-3', *GABRA2*_rev 5'-AGAGCCATCAGGAGCAACCTGT-3';

*PCDH11X*_for 5'-CCAATGAAGGAGGTTGTGCGATC-3', *PCDH11X*_rev 5'-TGAGAGCCTTCTGGCAGGTGAA-3';

*KHDRBS2*_for 5'-GCTTGGACCAAGAGGAAACTCC-3'; *KHDRBS2*_rev 5'-CAAGTGGGCATATTTGGCTTCCC-3';

*PLCXD3*_for 5'-CCTCACCAATTTAGCCATTCCAG-3', *PLCXD3*_rev 5'-GCCACAGTTCCAAACACAGAGAC-3'

*BAX*_for 5'-TCAGGATGCGTCCACCAAGAAG-3', *BAX*_rev 5'-TGTGTCCACGGCGGCAATCATC-3';

*CDKN1A*_for 5'-AGGTGGACCTGGAGACTCTCAG-3', *CDKN1A*_rev 5'-TCCTCTTGGAGAAGATCAGCCG-3';

*GADD45A*_for 5'-CTGGAGGAAGTGCTCAGCAAAG-3', *GADD45A*_rev 5'-AGAGCCACATCTCTGTCGTCGT-3';

*ITPR1*_for 5'-GTGACAGGAAACATGCAGACTCG-3', *ITPR1*_rev 5'-CAGCAGTTGCACAAAGACAGGC-3';

*TNFRSF10B*_for 5'-AGCACTCACTGGAATGACCTCC-3', *TNFRSF10B*_rev 5'-GTGCCTTCTTCGCACTGACACA-3';

*CAMK2D*_for 5'-ACACGGTGACTCCTGAAGCCAA-3', *CAMK2D*_rev 5'-GTCTCCTGTCTGTGCATCATGG-3';

*SQSTM1*_for 5'-TGTGTAGCGTCTGCGAGGGAAA-3', *SQSTM1*_rev 5'-AGTGTCCGTGTTTCACCTTCCG-3';

*MDM2*_for 5'-TGTTTGGCGTGCCAAGCTTCTC-3', *MDM2*_rev 5'-CACAGATGTACCTGAGTCCGATG-3';

*18s*_for 5'-AGTCCCTGCCCTTTGTACACA-3', *18s*_rev 5'-GATCCGAGGGCCTCACTAAAC-3'.

PCR conditions were as follows: 10 min at 95°C, 40 cycles of 10 sec at 95°C, 30 sec at 60°C, and 30 sec at 72°C, followed by melting curve stage of 1 min at 60°C and 15 sec at 95°C. Relative expression levels were calculated using the $2^{-\Delta\Delta Ct}$ method as previously described [6].

Bulk RNA-sequencing

QIAGEN kit extracted and purified RNA was submitted for bulk RNA-sequencing at the Columbia Genome Center using an Illumina NovaSeq 6000. Each sample was multiplexed and yielded paired-end 100bp reads. Real-Time Aanalysis (RTA, Illumina) was performed for base calling and bcl2fastq2 for converting BCL to fastq format with adaptor trimming. Transcriptomes were

pseudo-aligned to kallisto index (Ensembl v96, Human:GRCh38.p12) using kallisto. Differentially expressed genes (DEG) analysis was performed using the DESeq2 package in R [7]. Pathways of DEGs were identified using pathfindR and clusterProfiler packages in R as previously described [15, 16]. Heatmaps were generated as previously described using R to rescale RNA counts in between 0 and 1 and GraphPad Prism9 to plot data [12]. APP^{V717I} clone data were combined to represent RNA expression levels of APP^{V717I} cells and compared to their isogenic control. DEGs were filtered by RNA counts that have standard deviation less than 0.6 between two APP^{V717I} clones to minimize effects that are not induced by genetic changes.

snRNA-seq data analysis

In each sample run (batch), Cellranger v3 was used to map sequenced transcripts to a dual reference for human (GRCh38) and mouse (mm10). Cellbender https://www.biorxiv.org/content/10.1101/791699v1 was used to identify and remove background from Cellranger output "raw_feature_bc_matrix", subsequently only cells designated with detected cell barcodes from "filtered_feature_bc_matrix" were used for single nuclei data analysis using Seurat v4.0.3.

For grafted neuron single nuclei, we performed cell integration on Batch sequencing runs using harmony (https://www.nature.com/articles/s41592-019-0619-0) and resolved UMAP layout with 15 Principal Components. Two batches of snRNAseq were conducted and corrected in the analysis: batch 1: Ctrl 1-2 and APP^{V717I} 1-3; batch 2: Ctrl 3-6 and APP^{V717I} 4-6.

For each group, only genes expressed in at least 10 copies per cell were kept. Groups were merged into one data object and percentage of human and mouse mitochondrial related genes were assessed, percentage of human and mouse ribosomal related genes were evaluated after the removal of mitochondrial related genes for human and mouse. Merged data object was

filtered, keeping only cells with less than 5% mitochondrial related genes, more than 1000 and less than 25,000 unique mapped transcripts. For normalization of merged data object, Seurat function “SCTransform” was used to identify 3000 variable genes in all cells and regress out UMI counts, percent of mitochondrial related genes and percent of ribosomal related genes. 50 principal components and 20 neighbors were used to resolve PC space, UMAP space and cell clusters. Seurat function “FindAllMarkers” was used to find gene markers for cell clusters using 3000 variable genes identified by “SCTransform”.

For assessment whether a cell cluster consisted of predominantly human cells or mouse cells, gene markers for cell clusters were screened for human genes (GRCh38-GENE), mouse genes (mm10---Gene) or mix of human and mouse cells (GRCh38-GENE and mm10---Gene). First round of assessment separated human cell clusters and mouse cell clusters, enabling subsetting of data objects into human cells or mouse cells. Subsequent re-processing of human data object and mouse data object with re-normalization, re-clustering and screening of gene markers for cell clusters displayed emergence of mixed cell clusters in both human and mouse data objects. To clean up the data objects, cell clusters of mouse cells were removed from human data object and human cells were removed from mouse data object. The data objects were again re-processed, re-normalized, re-clustered and screened for gene markers. Procedure of screening and removal of mixed cell clusters was repeated until no mixed cell clusters were observed, 3 iterations were performed for human data object and 4 iterations for mouse data object. In human cell data object, two main cell types were observed, neurons and astrocytes. Subsetting of cell types into human neuron data object, human astrocyte data object further required re-processing, re-normalizing, re-clustering, assessment for gene markers and removal of cell clusters of mouse cells, 2 iterations were performed for each data object to obtain clean human cell type data objects.

Similar action was taken for cleaning up subsets of mouse cell type data objects with re-processing, re-normalization, re-clustering, gene marker assessment and removal of cell clusters of human cells, 2 to 3 iterations were performed for each mouse major cell type.

Additional action was taken to identify doublet/mixed cell type clusters in mouse cell types. Assessment for gene markers representing doublet/mix cell clusters contained typical markers for two major mouse cell types (e.g. astrocyte/neuron, neuron/oligodendrocyte, microglia/neuron, astrocyte/oligodendrocyte, microglia/oligodendrocyte), and these cell clusters were removed when observed in mouse cell type data objects.

The reprocessed and cleaned data objects were remerged, first, human cell type to human cell type, mouse cell type to mouse cell type, and second, re-merged human cell types to re-merged mouse cell types making top level cell type data object with human and mouse cells.

Rebuilding no prefix data objects for Human graft cells and Mouse host cells, the Human gene "GRCh38-"prefix and mouse gene "mm10---"prefix were removed by reprocessing the merged mouse host and human graft data object. For human graft cells, the counts matrix was extracted, rows with "mm10---" prefix were removed, and the human data object was re-normalized with Seurat function "SCTransform". For humanizing mouse host cells, the counts matrix was extracted, rows with "GRCh38-" prefix were removed and the mouse gene names were converted to equivalent human genes by using the R package biomaRt, the mouse data object was re-normalized with Seurat function "SCTransform". For both data objects, 30 principal components and 20 neighbors were used to resolve PC space, UMAP space and cell clusters. For subsets of neurons or astrocytes, UMAP dimensions were determined by the turning point of "ElbowPlot" and "RunUMAP" and "DimPlot" functions were employed to generate UMAPs.

Differential gene expression analysis

For differential gene expression analysis in subset of either human grafted neurons or human grafted astrocytes, Seurat function "FindAllMarkers" was used to find gene markers for defined cell clusters using 3000 variable genes identified by SCTransform with set parameters for "logfc.threshold = 0.15" and "min.cells.feature = 10". Genes that have adjusted p-value above 0.05 are considered as DEGs. Pathways of DEGs were identified using pathfindR and clusterProfiler packages in R as previously described [15, 16]. Heatmaps were generated as previously described using R to rescale RNA counts in between 0 and 1 and GraphPad Prism9 to plot data [12]. DEGs of *in vitro* differentiated neurons and *in vivo* grafts were compared with DEGs of neurons from sn-Seq analysis of human AD brains in published literature [2, 8] and an online database of GWAS catalog in AD [https://www. ebi. ac. uk/ gwas/] [14] Genes that have adjusted p-value below 0.05 are considered DEGs. The Venn diagram was generated using an online Bioinformatics&Evolutionary Genomics tool (https://bioinformatics.psb.ugent.be/webtools/Venn/).

Supplemental References

1 Bankhead P, Loughrey MB, Fernández JA, Dombrowski Y, McArt DG, Dunne PD, McQuaid S, Gray RT, Murray LJ, Coleman HG (2017) QuPath: Open source software for digital pathology image analysis. Scientific reports 7: 1-7

2 Cain A, Taga M, McCabe C, Hekselman I, White CC, Green G, Rozenblatt-Rosen O, Zhang F, Yeger-Lotem E, Bennett DA (2020) Multi-cellular communities are perturbed in the aging human brain and with Alzheimer’s disease. BioRxiv:

3 Ehrlich M, Hallmann AL, Reinhardt P, Arauzo-Bravo MJ, Korr S, Ropke A, Psathaki OE, Ehling P, Meuth SG, Oblak ALet al (2015) Distinct Neurodegenerative Changes in an Induced Pluripotent Stem Cell Model of Frontotemporal Dementia Linked to Mutant TAU Protein. Stem Cell Reports 5: 83-96 Doi S2213-6711(15)00182-4 [pii] 10.1016/j.stemcr.2015.06.001

4 Goncalves MD, Hwang S-K, Pauli C, Murphy CJ, Cheng Z, Hopkins BD, Wu D, Loughran RM, Emerling BM, Zhang G (2018) Fenofibrate prevents skeletal muscle loss in mice with lung cancer. Proceedings of the National Academy of Sciences 115: E743-E752

5 Lim RG, Al-Dalahmah O, Wu J, Gold MP, Reidling JC, Tang G, Adam M, Dansu DK, Park H-J, Casaccia P (2022) Huntington disease oligodendrocyte maturation deficits revealed by single-nucleus RNAseq are rescued by thiamine-biotin supplementation. Nature Communications 13: 7791

6 Livak KJ, Schmittgen TD (2001) Analysis of relative gene expression data using real-time quantitative PCR and the 2− ΔΔCT method. methods 25: 402-408

7 Love MI, Huber W, Anders S (2014) Moderated estimation of fold change and dispersion for RNA-seq data with DESeq2. Genome biology 15: 1-21

8 Mathys H, Davila-Velderrain J, Peng Z, Gao F, Mohammadi S, Young JZ, Menon M, He L, Abdurrob F, Jiang X (2019) Single-cell transcriptomic analysis of Alzheimer’s disease. Nature 570: 332-337

9 Nguyen TT, Shang E, Shu C, Kim S, Mela A, Humala N, Mahajan A, Yang HW, Akman HO, Quinzii CM (2021) Aurora kinase A inhibition reverses the Warburg effect and elicits unique metabolic vulnerabilities in glioblastoma. Nature communications 12: 5203

10 Nguyen TTT, Ishida CT, Shang E, Shu C, Torrini C, Zhang Y, Bianchetti E, Sanchez-Quintero MJ, Kleiner G, Quinzii CM (2019) Activation of LXR β inhibits tumor respiration and is synthetically lethal with Bcl-xL inhibition. EMBO Molecular Medicine 11: e10769

11 Pang Z, Zhou G, Ewald J, Chang L, Hacariz O, Basu N, Xia J (2022) Using MetaboAnalyst 5.0 for LC–HRMS spectra processing, multi-omics integration and covariate adjustment of global metabolomics data. Nature Protocols 17: 1735-1761

12 Qu W, Jeong A, Zhong R, Thieschafer JS, Gram A, Li L (2023) Deletion of Small GTPase H-Ras Rescues Memory Deficits and Reduces Amyloid Plaque-Associated Dendritic Spine Loss in Transgenic Alzheimer’s Mice. Molecular Neurobiology 60: 495-511

13 Qu W, Johnson A, Kim JH, Lukowicz A, Svedberg D, Cvetanovic M (2017) Inhibition of colony-stimulating factor 1 receptor early in disease ameliorates motor deficits in SCA1 mice. Journal of neuroinflammation 14: 1-11

14 Sollis E, Mosaku A, Abid A, Buniello A, Cerezo M, Gil L, Groza T, Gunes O, Hall P, Hayhurst Jet al (2023) The NHGRI-EBI GWAS Catalog: knowledgebase and deposition resource. Nucleic Acids Res 51: D977-D985 Doi 10.1093/nar/gkac1010

15 Ulgen E, Ozisik O, Sezerman OU (2019) pathfindR: An R Package for Comprehensive Identification of Enriched Pathways in Omics Data Through Active Subnetworks. Front Genet 10: 858 Doi 10.3389/fgene.2019.00858

16 Wu T, Hu E, Xu S, Chen M, Guo P, Dai Z, Feng T, Zhou L, Tang W, Zhan L (2021) clusterProfiler 4.0: A universal enrichment tool for interpreting omics data. The Innovation 2: 100141
