# Supplementary material for: Xenografted human iPSC-derived neurons with the familial Alzheimer’s disease APPV717I mutation reveal dysregulated transcriptome signatures linked to synaptic function and implicate LINGO2 as a disease signaling mediator
Source: Acta Neuropathol. 2024 Jun 25;147(1):107. doi: 10.1007/s00401-024-02755-5 (PMC11199265; doi:10.1007/s00401-024-02755-5)
Supplement: Supplementary file 2 — Supplementary file2 (PDF 13287 kb) [file 401_2024_2755_MOESM2_ESM.pdf]

Supplemental Figures

Xenografted human iPSC-derived neurons with the familial Alzheimer's disease *APP*^{V717I} mutation reveal dysregulated transcriptome signatures linked to synaptic function and implicate LINGO2 as a disease signaling mediator

Wenhui Qu, Matti Lam, Julie J. McInvale, Jason A. Mares, Sam Kwon, Nelson Humala, Aayushi Mahajan, Trang Nguyen, Kelly A. Jakubiak, Jeong-Yeon Mun, Thomas G. Tedesco, Osama Al-Dalahmah, Syed A. Hussaini, Andrew A. Sproul, Markus D. Siegelin, Philip L. De Jager, Peter Canoll, Vilas Menon, and Gunnar Hargus

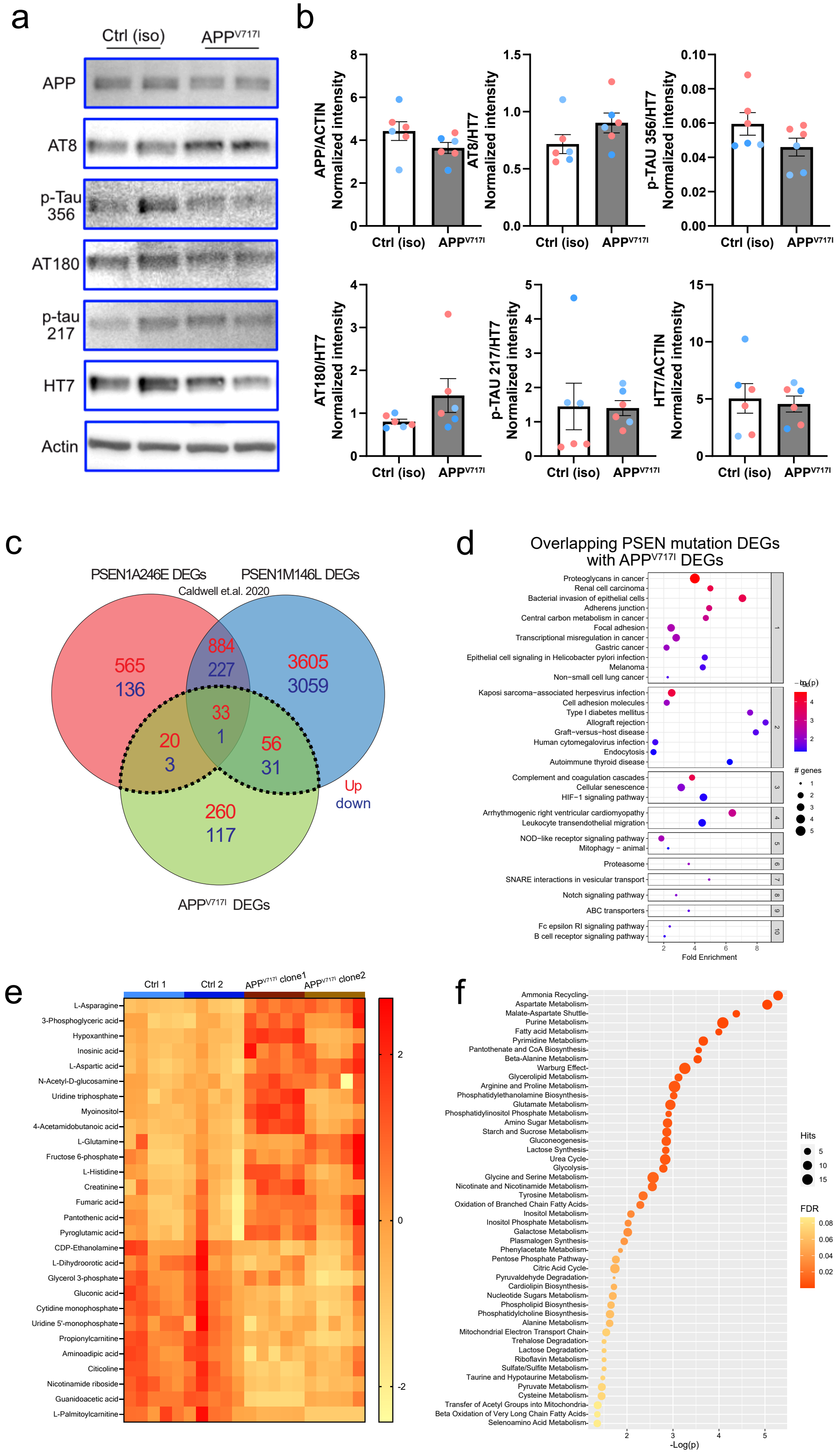
a
Ctrl (iso)
APPV717I
APP
AT8
p-Tau 356
AT180
p-tau 217
HT7
Actin
b
APP/ACTIN Normalized intensity
AT8/HT7 Normalized intensity
p-TAU 356/HT7 Normalized intensity
AT180/HT7 Normalized intensity
p-TAU 217/HT7 Normalized intensity
HT7/ACTIN Normalized intensity
Ctrl (iso)
APPV717I
c
PSEN1A246E DEGs
PSEN1M146L DEGs
Caldwell et.al. 2020
565
136
884
227
3605
3059
33
1
20
3
56
31
260
117
Up
down
APPV717I DEGs
d
Overlapping PSEN mutation DEGs with APPV717I DEGs
Proteoglycans in cancer
Renal cell carcinoma
Bacterial invasion of epithelial cells
Adherens junction
Central carbon metabolism in cancer
Focal adhesion
Transcriptional misregulation in cancer
Gastric cancer
Epithelial cell signaling in Helicobacter pylori infection
Melanoma
Non−small cell lung cancer
Kaposi sarcoma−associated herpesvirus infection
Cell adhesion molecules
Type I diabetes mellitus
Allograft rejection
Graft−versus−host disease
Human cytomegalovirus infection
Endocytosis
Autoimmune thyroid disease
Complement and coagulation cascades
Cellular senescence
HIF−1 signaling pathway
Arrhythmogenic right ventricular cardiomyopathy
Leukocyte transendothelial migration
NOD−like receptor signaling pathway
Mitophagy − animal
Proteasome
SNARE interactions in vesicular transport
Notch signaling pathway
ABC transporters
Fc epsilon RI signaling pathway
B cell receptor signaling pathway
Fold Enrichment
genes
e
Ctrl 1
Ctrl 2
APPV717I clone1
APPV717I clone2
L-Asparagine
3-Phosphoglyceric acid
Hypoxanthine
Inosinic acid
L-Aspartic acid
N-Acetyl-D-glucosamine
Uridine triphosphate
Myoinositol
4-Acetamidobutanoic acid
L-Glutamine
Fructose 6-phosphate
L-Histidine
Creatinine
Fumaric acid
Pantothenic acid
Pyroglutamic acid
CDP-Ethanolamine
L-Dihydroorotic acid
Glycerol 3-phosphate
Gluconic acid
Cytidine monophosphate
Uridine 5'-monophosphate
Propionylcarnitine
Aminoadipic acid
Citicoline
Nicotinamide riboside
Guanidoacetic acid
L-Palmitoylcarnitine
f
Ammonia Recycling
Aspartate Metabolism
Malate-Aspartate Shuttle
Purine Metabolism
Fatty acid Metabolism
Pyrimidine Metabolism
Pantothenate and CoA Biosynthesis
Beta-Alanine Metabolism
Warburg Effect
Glycerolipid Metabolism
Arginine and Proline Metabolism
Phosphatidylethanolamine Biosynthesis
Glutamate Metabolism
Phosphatidylinositol Phosphate Metabolism
Amino Sugar Metabolism
Starch and Sucrose Metabolism
Gluconeogenesis
Lactose Synthesis
Urea Cycle
Glycolysis
Glycine and Serine Metabolism
Nicotinate and Nicotinamide Metabolism
Tyrosine Metabolism
Oxidation of Branched Chain Fatty Acids
Inositol Metabolism
Inositol Phosphate Metabolism
Galactose Metabolism
Plasmalogen Synthesis
Phenylacetate Metabolism
Pentose Phosphate Pathway
Citric Acid Cycle
Pyruvaldehyde Degradation
Cardiolipin Biosynthesis
Nucleotide Sugars Metabolism
Phospholipid Biosynthesis
Phosphatidylcholine Biosynthesis
Alanine Metabolism
Mitochondrial Electron Transport Chain
Trehalose Degradation
Lactose Degradation
Riboflavin Metabolism
Sulfate/Sulfite Metabolism
Taurine and Hypotaurine Metabolism
Pyruvate Metabolism
Cysteine Metabolism
Transfer of Acetyl Groups into Mitochondria
Beta Oxidation of Very Long Chain Fatty Acids
Selenoamino Acid Metabolism
-Log(p)
Hits
FDR

Figure S1: Expression of p-tau and APP, comparative DEG analysis, and mass spectrometry for polar metabolites in APP^{V717I} and Ctrl neurons.

(a-b) Representative western blotting images **(a)** and quantifications **(b)** showing similar p-tau levels, total APP expression, and total tau expression in cultured APP^{V717I} and Ctrl neurons. Blue and red dots represent the two different cell clones and each dot represents an independent differentiation.

(c) Venn diagram comparing *in vitro* DEGs of this study with published DEGs from neurons derived from PSEN1^{A246E} and PSEN1^{M146L} mutations (PMID: 33188013).

(d) Pathway analysis derived from common DEGs in APP^{V717I} neurons and PSEN1^{A246E} and PSEN1^{M146L} neurons (black circle heighted in (c)) showing convergent dysregulated pathways in different fAD neurons.

(e) Heatmap showing the abundance of 28 different polar metabolites identified through mass spectrometry that are similarly expressed in the two APP^{V717I} clones but are significantly different from Ctrls.

(f) Quantitative pathway enrichment analysis of metabolic profiles showing the metabolic pathways that are altered in APP^{V717I} neurons.

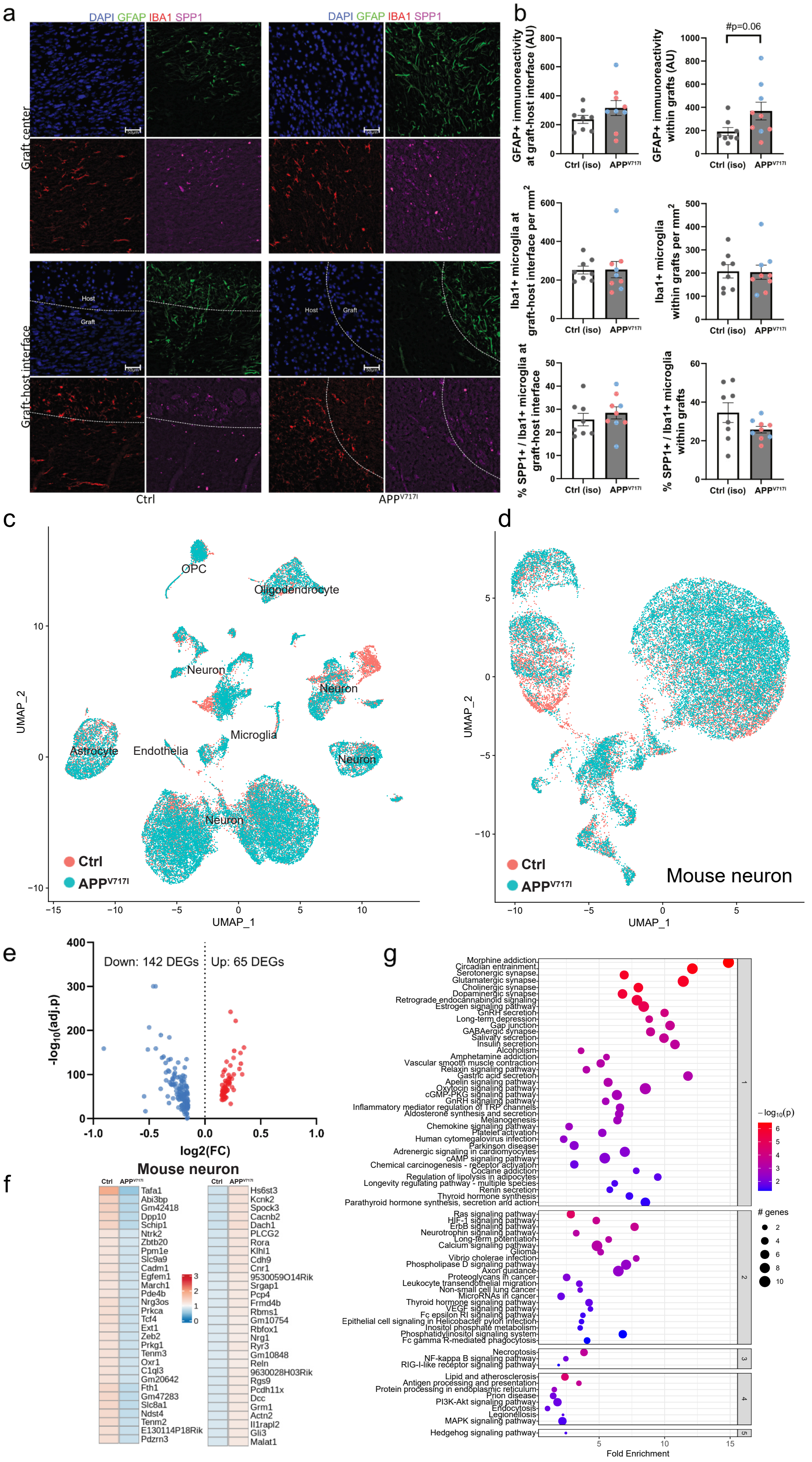
a
DAPI GFAP IBA1 SPP1
DAPI GFAP IBA1 SPP1
Graft center
Graft-host interface
Host
Graft
Host
Graft
Ctrl
APPV717I
b
GFAP+ immunoreactivity at graft-host interface (AU)
GFAP+ immunoreactivity within grafts (AU)
#p=0.06
Iba1+ microglia at graft-host interface per mm2
Iba1+ microglia within grafts per mm2
% SPP1+ / Iba1+ microglia at graft-host interface
% SPP1+ / Iba1+ microglia within grafts
Ctrl (iso)
APPV717I
c
OPC
Oligodendrocyte
Neuron
Neuron
Microglia
Astrocyte
Endothelia
Neuron
Neuron
Ctrl
APPV717I
UMAP_1
UMAP_2
d
Ctrl
APPV717I
Mouse neuron
UMAP_1
UMAP_2
e
Down: 142 DEGs
Up: 65 DEGs
-log10(adj.p)
log2(FC)
f
Mouse neuron
Ctrl
APPV717I
Tafa1
Abi3bp
Gm42418
Dpp10
Schip1
Ntrk2
Zbtb20
Ppm1e
Slc9a9
Cadm1
Egfem1
March1
Pde4b
Nrg3os
Prkca
Tcf4
Ext1
Zeb2
Prkg1
Tenm3
Oxr1
C1ql3
Gm20642
Fth1
Gm47283
Slc8a1
Ndst4
Tenm2
E130114P18Rik
Pdzrn3
Hs6st3
Kcnk2
Spock3
Cacnb2
Dach1
PLCG2
Rora
Klhl1
Cdh9
Cnr1
9530059O14Rik
Srgap1
Pcp4
Frmd4b
Rbms1
Gm10754
Rbfox1
Nrg1
Ryr3
Gm10848
Reln
9630028H03Rik
Rgs9
Pcdh11x
Dcc
Grm1
Actn2
Il1rapl2
Gli3
Malat1
g
Morphine addiction
Circadian entrainment
Serotonergic synapse
Glutamatergic synapse
Cholinergic synapse
Dopaminergic synapse
Retrograde endocannabinoid signaling
Estrogen signaling pathway
GnRH secretion
Long-term depression
Gap junction
GABAergic synapse
Salivary secretion
Insulin secretion
Alcoholism
Amphetamine addiction
Vascular smooth muscle contraction
Relaxin signaling pathway
Gastric acid secretion
Apelin signaling pathway
Oxytocin signaling pathway
cGMP-PKG signaling pathway
GnRH signaling pathway
Inflammatory mediator regulation of TRP channels
Aldosterone synthesis and secretion
Melanogenesis
Chemokine signaling pathway
Platelet activation
Human cytomegalovirus infection
Parkinson disease
Adrenergic signaling in cardiomyocytes
cAMP signaling pathway
Chemical carcinogenesis - receptor activation
Cocaine addiction
Regulation of lipolysis in adipocytes
Longevity regulating pathway - multiple species
Renin secretion
Thyroid hormone synthesis
Parathyroid hormone synthesis, secretion and action
Ras signaling pathway
HIF-1 signaling pathway
ErbB signaling pathway
Neurotrophin signaling pathway
Long-term potentiation
Calcium signaling pathway
Glioma
Vibrio cholerae infection
Phospholipase D signaling pathway
Axon guidance
Proteoglycans in cancer
Leukocyte transendothelial migration
Non-small cell lung cancer
MicroRNAs in cancer
Thyroid hormone signaling pathway
VEGF signaling pathway
Fc epsilon RI signaling pathway
Epithelial cell signaling in Helicobacter pylori infection
Inositol phosphate metabolism
Phosphatidylinositol signaling system
Fc gamma R-mediated phagocytosis
Necroptosis
NF-kappa B signaling pathway
RIG-I-like receptor signaling pathway
Lipid and atherosclerosis
Antigen processing and presentation
Protein processing in endoplasmic reticulum
Prion disease
PI3K-Akt signaling pathway
Endocytosis
Legionellosis
MAPK signaling pathway
Hedgehog signaling pathway
Fold Enrichment
−log10(p)
genes

Figure S2: Grafted APP^{V717I} neurons do not alter a glial response in mouse brains but shift the transcriptome profile of mouse neurons.

(a-b) Immunostainings (a) and quantification (b) of GFAP immunoreactivity in astrocytes as well as Iba1^{+} and SPP1^{+} microglia in the graft center and at the graft-host interface. Nuclei are counterstained with DAPI.

(c) UMAP of sn-RNAseq of mouse cells within and around micro-dissected APP^{V717I} and Ctrl grafts showing the different cell types in the mouse brains.

(d) UMAP of mouse neurons showing a change in the transcriptome profile in the APP^{V717I} group.

(e) Volcano plot showing up- and downregulated DEGs in mouse neurons within and adjacent to APP^{V717I} grafts.

(f) Heatmaps of the top 30 up- and downregulated DEGs in mouse neurons in the APP^{V717I} versus Ctrl group.

(g) Pathway enrichment analysis demonstrating the cellular pathways in mouse neurons that have been altered by the transplantation of human APP^{V717I} cells, including several synaptic function-related pathways (glutamatergic synapse, GABAergic synapse, long-term potentiation, calcium signaling, and long-term depression), small GTPase mediated pathways, axon guidance, and the MAPK signaling pathway.

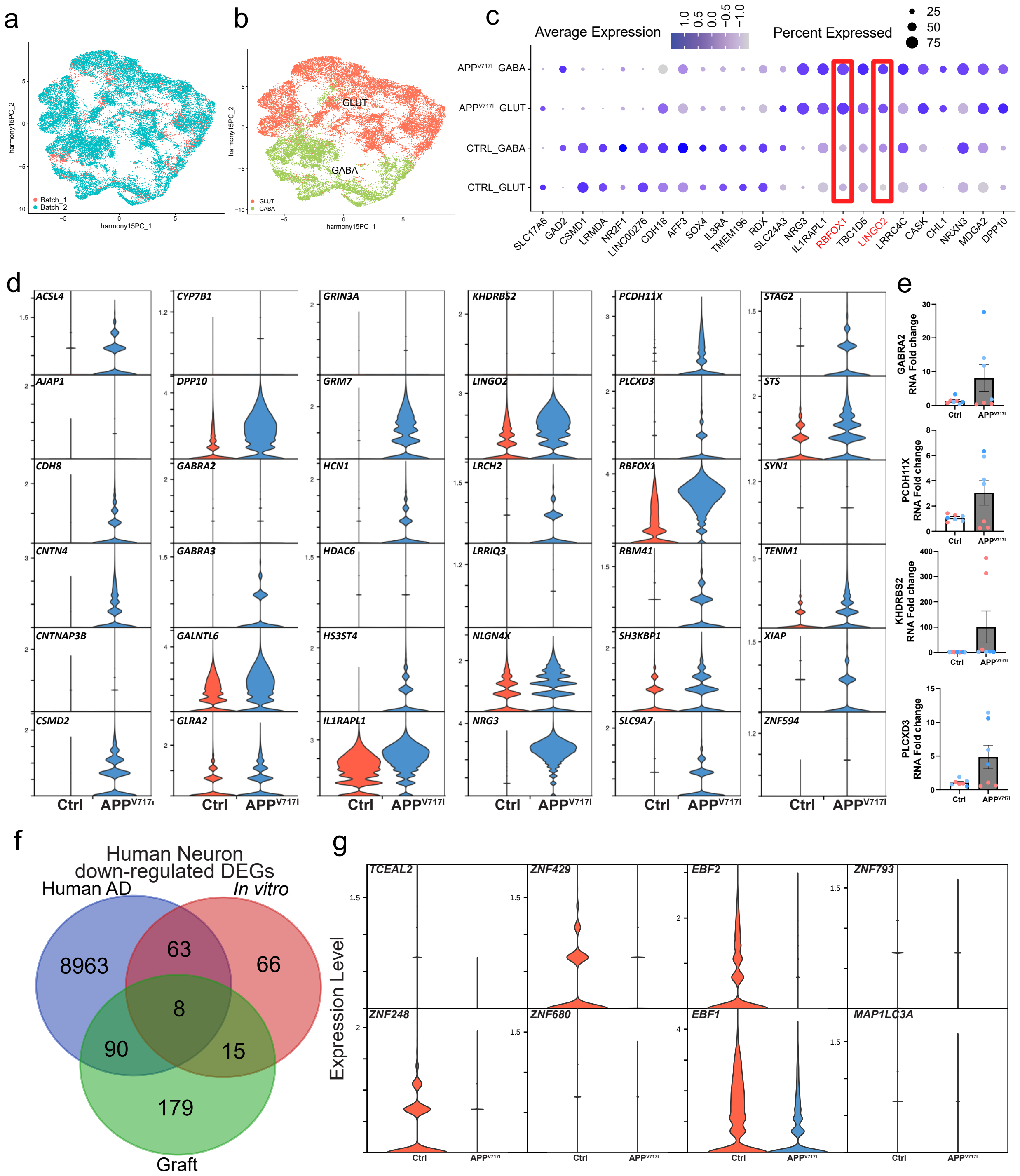
a
b
c
d
e
f
g
Average Expression
Percent Expressed
Human Neuron
down-regulated DEGs
Human AD
In vitro
Graft
8963
63
66
8
90
15
179
Expression Level
GLUT
GABA
Ctrl
APPV717I

Figure S3: Additional characterization of transplanted human APP^{V717I} neurons.

(a) UMAP plot showing nuclei of APP^{V717I} and Ctrl nuclei color coded by batch.

Nuclei isolation and snRNAseq were conducted in two batches and no separation between two batches was observed.

(b) UMAP plot showing that neurons can be subclustered into glutamatergic and GABAergic neurons.

(c) Dot plot highlighting genes that are altered in grafted APP^{V717I} neurons, both in glutamatergic and GABAergic neurons.

(d) Additional violin plots related to Figure 4d showing expression levels of indicated genes in grafted APP^{V717I} and Ctrl neurons.

(e) RT-qPCR results for assessment of the expression of selected genes in cultured APP^{V717I} versus Ctrl neurons, related to Figure 4e.

(f) Venn diagram showing the overlap of downregulated DEGs in human postmortem AD neurons, cultured APP^{V717I} neurons and grafted APP^{V717I} neurons.

(g) Violin plots demonstrating low expression levels of the 8 overlapping DEGs in grafted neurons.

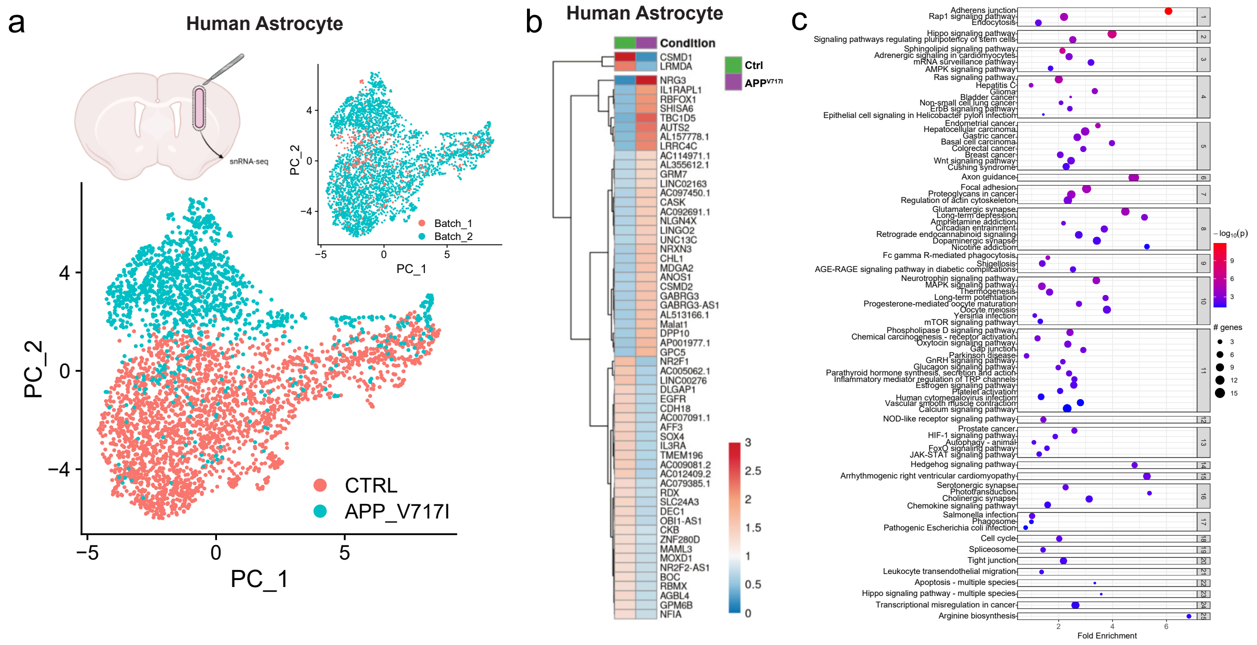

Figure S4: Characterization of transplanted human APP^{V717I} astrocytes.

(a) UMAP plot of grafted human astrocytes showing clear separation of transcriptome profiles in APP^{V717I} astrocytes compared to Ctrl astrocytes.

(b) Top 30 up- or downregulated DEGs of grafted human APP^{V717I} astrocytes.

(c) Pathway enrichment analysis of DEGs in grafted APP^{V717I} astrocytes demonstrating indicated perturbed cellular pathways, including glutamatergic synapse, calcium signaling, axon guidance and Rap and Ras signaling pathways, that play importvant roles in the pathogenesis of AD.

12 months post injection

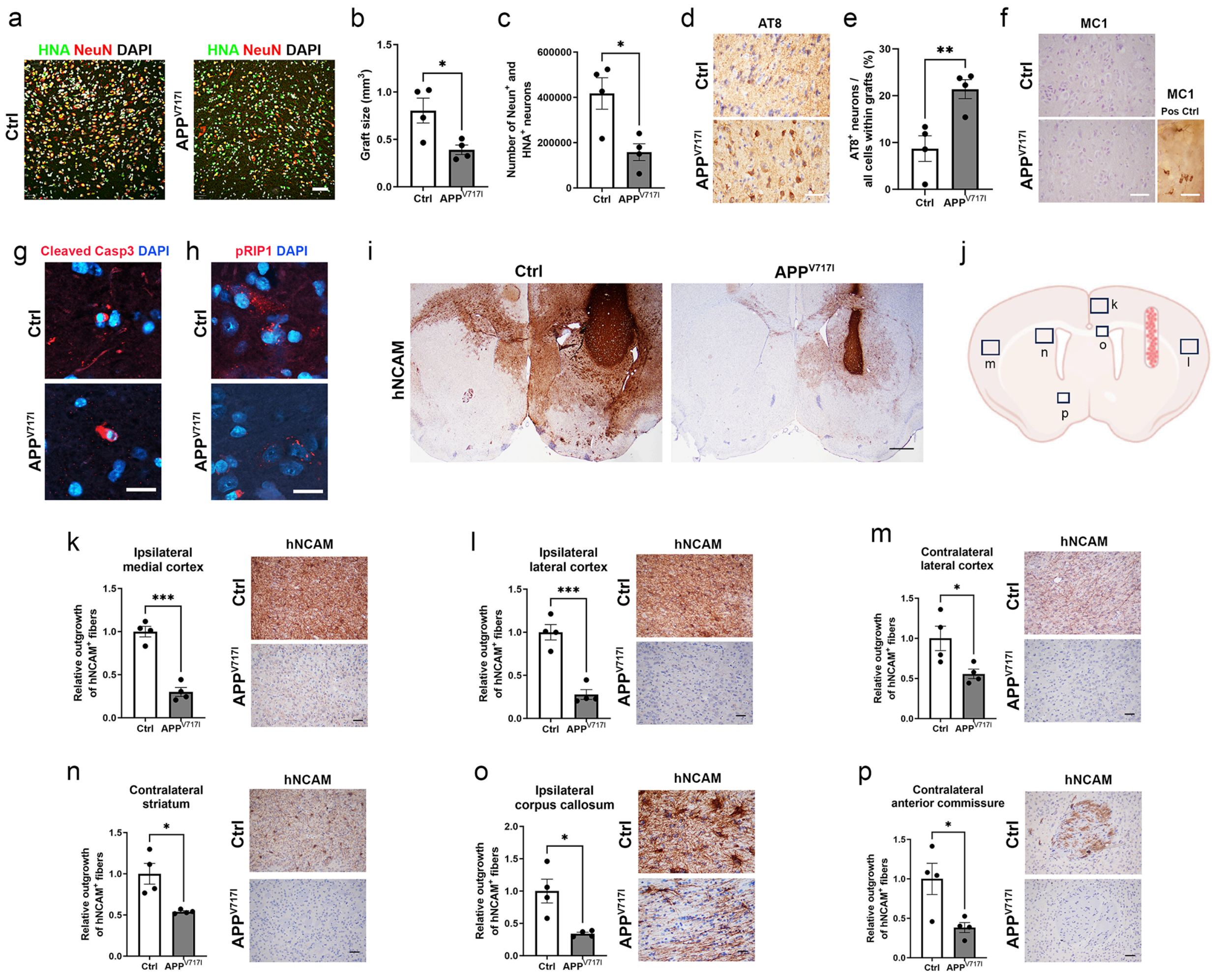

Figure S5: Characterization of APP^{V717I} and Ctrl grafts 12 months after cell injection.

(a) Immunostained grafts 12 months after injection of APP^{V717I} or Ctrl NPCs into the cortex and striatum of adult mice using antibodies for NeuN and human nuclear antigen (HNA).

(b-c) Quantification of the graft size (b) and of the number of grafted $Neun^{+}$ and HNA^{+} neurons (c) 12 months after cell injection.

(d-e) Immunostainings of APP^{V717I} or Ctrl grafts for p-tau (AT8) (d) with quantification (e). Scale bar, 50µm

(f) Immunostainings of APP^{V717I} or Ctrl grafts for MC1. Scale bars, 50µm

(g-h) Immunostainings of APP^{V717I} or Ctrl grafts for cleaved Caspase 3 (g) or pRIP1 (h). Nuclei were counterstained with DAPI. Scale bars, 20µm.

(i) Representative images of hNCAM-stained brains with APP^{V717I} or Ctrl neural grafts 12 months after cell injection.

(j) Schematic illustration of APP^{V717I} and Ctrl neural grafts 12 months after cell injection with areas chosen for quantification of hNCAM+ fibers in the ipsilateral and contralateral hemisphere, provided in panels k-p.

(k-p) Quantification of the relative abundance of $hNCAM^{+}$ fibers in the ipsilateral medial cortex (k), ipsilateral lateral cortex (l), contralateral lateral cortex (m), contralateral striatum (n), ipsilateral corpus callosum (o) and contralateral anterior commissure (p). Representative images of $hNCAM^{+}$ fibers are shown for each region.

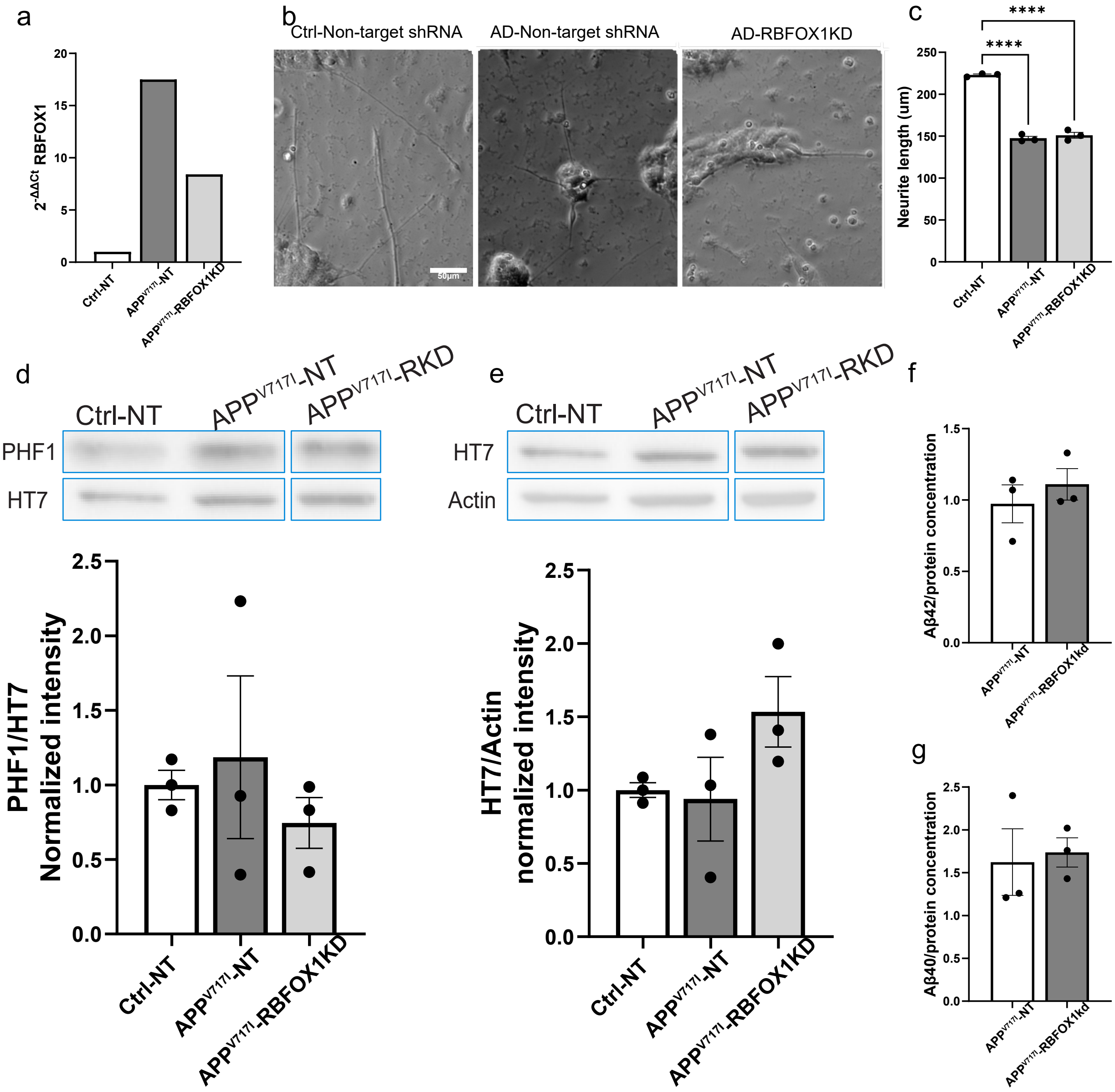

Figure S6: RBFOX1 knockdown has limited effect on APP^{V717I} cells.

(a) RT-qPCR for *RBFOX1* confirming *RBFOX1* knockdown in APP^{V717I} neurons.

(b-c) Representative images (c) and quantification (c) of neurite outgrowth showing that RBFOX1 knockdown does not affect neurite outgrowth in APP^{V717I} neurons.

(d-e) Western blot images and quantification demonstrating that RBFOX1 downregulation does not affect p-tau or total tau levels.

(f-g) ELISA for Aβ_{42} (f) and Aβ_{40} (g) on conditioned media of APP^{V717I} neurons with and without RBFOX1 KD showing that RBFOX1 downregulation does not affect Aβ levels.

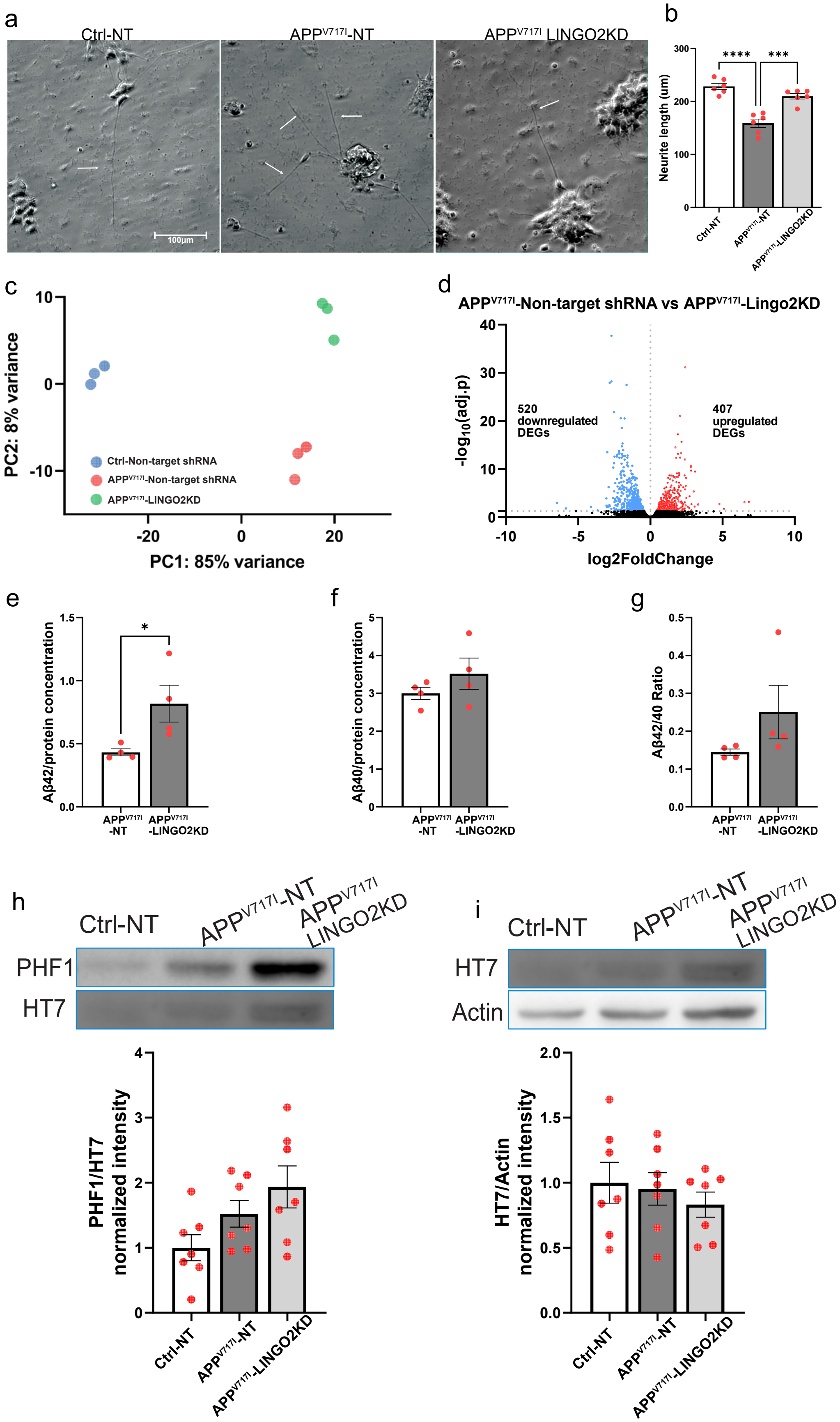

a
Ctrl-NT
APPV717I-NT
APPV717I LINGO2KD
100μm
b
Neurite length (um)
****
***
Ctrl-NT
APPV717I-NT
APPV717I-LINGO2KD
c
PC2: 8% variance
PC1: 85% variance
Ctrl-Non-target shRNA
APPV717I-Non-target shRNA
APPV717I-LINGO2KD
d
APPV717I-Non-target shRNA vs APPV717I-Lingo2KD
-log10(adj.p)
log2FoldChange
520 downregulated DEGs
407 upregulated DEGs
e
Aβ42/protein concentration
*
APPV717I -NT
APPV717I -LINGO2KD
f
Aβ40/protein concentration
g
Aβ42/40 Ratio
h
Ctrl-NT
APPV717I-NT
APPV717I LINGO2KD
PHF1
HT7
PHF1/HT7 normalized intensity
i
HT7
Actin
HT7/Actin normalized intensity

Figure S7: The effect of LINGO2 knockdown is independent of Aβ or tau pathology.

(a-b) Representative images of neurite outgrowth measured five days after differentiation imaged under bright field (a) and quantifications (b).

(c) PCA plot highlighting the separation of transcriptome profiles in APP^{V717I}, APP^{V717I}-LINGO2KD and Ctrl cells.

(d) Volcano plot showing up- and downregulated DEGs in APP^{V717I}-LINGO2KD compared to APP^{V717I} cells.

(e-g) ELISA for $A\beta_{42}$ and $A\beta_{40}$ on conditioned media of APP^{V717I} neurons with and without LINGO2 KD showing increased $A\beta_{42}$ production in APP^{V717I}-LINGO2KD cells but no changes in $A\beta_{40}$ or $A\beta_{42}$/ $A\beta_{40}$ ratio.

(h-i) Western blot images and quantification demonstrating that LINGO2 downregulation does not affect p-tau or total tau levels.

One-way ANOVA with Tukey post-hoc test was used in panel b. Student t-test was used in panel e, *p<0.05, ***p<0.001, ****p<0.0001. Ctrl-NT and APP^{V717I}-NT: Ctrl and APP^{V717I} neurons transduced with non-target-control virus.
